# Dogs, but Not Wolves, Lose Their Sensitivity Toward Novelty With Age

**DOI:** 10.3389/fpsyg.2019.02001

**Published:** 2019-09-04

**Authors:** Christina Hansen Wheat, Wouter van der Bijl, Hans Temrin

**Affiliations:** ^1^Department of Zoology, Stockholm University, Stockholm, Sweden; ^2^Department of Zoology and Biodiversity Research Centre, University of British Columbia, Vancouver, BC, Canada

**Keywords:** fear, domestication, sensitive period, behavioral ontogeny, neophobia, dogs, wolves

## Abstract

Selection of behavioral traits holds a prominent role in the domestication of animals, and domesticated species are generally assumed to express reduced fear and reactivity toward novel stimuli compared to their ancestral species. However, very few studies have explicitly tested this proposed link between domestication and reduced fear responses. Of the limited number of studies experimentally addressing the alterations of fear during domestication, the majority has been done on canids. These studies on foxes, wolves, and dogs suggest that decreased expression of fear in domesticated animals is linked to a domestication-driven delay in the first onset of fearful behavior during early ontogeny. Thus, wolves are expected to express exaggerated fearfulness earlier during ontogeny compared to dogs. However, while adult dogs are less fearful toward novelty than adult wolves and wolf-dog hybrids, consensus is lacking on when differences in fear expression arise in wolves and dogs. Here we present the first extended examination of fear development in hand-raised dogs and European gray wolves, using repeated novel object tests from 6 to 26 weeks of age. Contrary to expectations, we found no evidence in support of an increase in fearfulness in wolves with age or a delayed onset of fear response in dogs compared to wolves. Instead, we found that dogs strongly reduced their fear response in the period between 6 and 26 weeks of age, resulting in a significant species difference in fear expression toward novelty from the age of 18 weeks. Critically, as wolves did not differ in their fear response toward novelty over time, the detected species difference was caused solely by a progressive reduced fear response in dogs. Our results thereby suggest that species differences in fear of novelty between wolves and dogs are not caused by a domestication-driven shift in the first onset of fear response. Instead, we suggest that a loss of sensitivity toward novelty with age in dogs causes the difference in fear expression toward novelty in wolves and dogs.

## Introduction

Humans have successfully domesticated a wide range of plants and animals and abundant evidence demonstrates how domesticated species express dramatically altered phenotypes compared to their wild counterparts (Driscoll et al., 2009). For animals, this includes changes in expression of a number of behaviors, including fear (Belyaev et al., 1985; Trut, 1999). Fear is a basic behavior in wild-living animal populations, as a timely and proper response to novelty (e.g., flight response versus exploration) has direct fitness consequences (Boissy, 1995; Weidenmayer, 2009). In contrast, strong fear responses and high reactivity toward novelty are undesirable traits in domesticated animals living in human-controlled environments (Leiner and Fendt, 2011), and selection for docility (i.e., tameness), and thus against fearfulness, was likely a key component in the successful domestication of animals (Belyaev et al., 1985; Trut, 1999). Consequently, it is generally assumed that domesticated species express reduced flight distances and reactivity toward novel stimuli (Zeder, 2012) compared to their ancestral species. However, though good evidence exists that cortisol secretion and brain structures associated with fear responses are significantly reduced in domesticated animals (Kruska, 1988; Trut et al., 2009), excessive fear behavior prevails in various domesticated species (Hemsworth et al., 1996), including rabbits (Csatádi et al., 2005), chickens (Jones and Waddington, 1992), dogs (Döring et al., 2009), and horses (Christensen et al., 2008). These discrepancies impair our understanding and expectations of how the expression of fear has changed during animal domestication and this shortcoming is further complicated by the fact that very few studies have explicitly tested the proposed link between domestication and reduced fear responses.

In wild populations appropriate fear responses are formed and modified throughout ontogeny, during which juvenile animals gradually combine individual experience and social information to develop the ability to discriminate between threatening and neutral stimuli (Scott and Fuller, 1965; Griffin, 2004; Weidenmayer, 2009). Ontogeny has been modified in several ways by domestication and compared to ancestral species, domesticated animals express altered developmental rates (Price, 1999; Dobney and Larson, 2006), a phenomenon known as heterochrony (Goodwin et al., 1997). Heterochrony has specifically been suggested to affect behavioral ontogeny by prolonging the sensitive period (Martin, 1978; Belyaev et al., 1985; Gariépy et al., 2001; Wilkins et al., 2014), an important period during behavioral development in which the juvenile animal is particularly sensitive to imprint on and form social bonds with conspecifics (Freedman et al., 1961; Scott, 1962; Scott and Fuller, 1965; Coppinger and Coppinger, 2001). During the sensitive period juvenile animals show increased exploratory behavior, as they readily approach novel stimuli and thereby learn about and socialize with their environment (Morrow et al., 2015). Importantly, the end of the sensitive period is marked by a progressive increase in fear and decreased exploration of novelty (Freedman et al., 1961; Belyaev et al., 1985). Based primarily on the findings in a long-term selection study on silver foxes (*Vulpes vulpes*), it has been suggested that domestication causes a shift in the sensitive period resulting in a delayed onset of fearful response in domesticated compared to non-domesticated animals (Belyaev et al., 1985; Trut et al., 2004; but see also Coppinger and Coppinger, 2001). While this might indicate that differences in fear expression between domesticated and non-domesticated animals arise already during early ontogeny, only a very limited body of studies have experimentally compared the ontogeny of fear in wild and domestic species under controlled conditions and with ambiguous results (Bilkó and Altbäcker, 2000; Lord, 2013). Therefore, it remains largely an open question whether the ontogeny of fear and the sensitive period have been altered by domestication.

The domestic dog (*Canis familiaris*) is an excellent study species when addressing questions about how domestication has affected behavioral ontogeny. Domestication of the dog from the gray wolf (*Canis lupus*) occurred at least 15,000 years ago (Driscoll et al., 2009), making the dog the first species to be domesticated. Studies of behavioral ontogeny in dogs have largely focused on the sensitive period, and fear of novelty in the dog puppy has traditionally been reported to manifest at 8 weeks of age and continually increase onward (Scott and Marston, 1950; Scott, 1958; Freedman et al., 1961; Scott and Fuller, 1965). However, recent evidence suggests that the development of fear might be highly breed-specific and subject to considerable variation (Morrow et al., 2015), thereby highlighting substantial gaps in our knowledge of the ontogeny of fear in dogs. In wolves, consensus on when fear behavior is established is lacking, with the onset of fearful response reported to occur as varied as 4–8 weeks of age across studies (Scott and Marston, 1950; Fentress, 1967; Wooply and Ginsburg, 1967; Fox, 1972; Zimen, 1987; Lord, 2013). The ambiguity of these wolf studies is further complicated by the fact that the majority of them were conducted over a short period of time and/or focused on isolated individuals or single litters, thereby limiting our ability to generalize from these findings. Additionally, a recent study found no difference in fear related behaviors and the latency to make contact with a novel object in 6 and 8 week old wolves and dogs (Marshall-Pescini et al., 2017), thereby suggesting that wolves might not express fear toward novelty at an earlier age than dogs. Thus, while adult wolves (Moretti et al., 2015) and wolf-dog hybrids (Hansen Wheat et al., 2018) are more fearful of novelty than dogs, the question of when during development species differences in fear expression are established remains unresolved. Furthermore, both juvenile and adult wolves explore and interact with novel objects more than similar aged dogs (Moretti et al., 2015; Marshall-Pescini et al., 2017), and adult dogs have been reported to be less likely to approach a novel object than wolves (Moretti et al., 2015). While these findings can be interpreted as dogs expressing less interest in the novel objects presented, and not fear, compared to wolves (Moretti et al., 2015), more studies are needed to tease these components apart and provide more detailed insight into how, and at which developmental stage, domestication has changed fear expression in wolves and dogs.

The lack of consensus across studies comparing wolves and dogs to uncover implications of domestication illustrates a fundamental challenge in this field, namely the combination of limited animal availability and the enormous effort necessary to hand-raise, socialize and test acquired animals. These challenges inherently lead to small sample sizes rarely exceeding *N* = 11 for wolves and *N* = 13 for dogs in contemporary studies where animals are hand-raised under identical conditions for species comparisons (Miklósi et al., 2003; Gácsi et al., 2005; Topál et al., 2005; Udell et al., 2008, 2012; Moretti et al., 2015; Range et al., 2015; Marshall-Pescini et al., 2017). Hand-raising wolves and dogs under similar conditions is imperative, as behavioral development is highly influenced by environmental factors (Zimen, 1987; Wilsson and Sundgren, 1998; Bray et al., 2017). Thus, because we heavily rely on these studies, with small sample sizes, to further increase our understanding of the domestication driven behavioral changes from wolf to dog, the importance of standardizing and reporting variations found across studies comparing wolves and dogs becomes critical.

Here we examined the development of fear toward novelty in wolves and dogs during the first 6 months of life, using standardized methods for both hand-raising, socializing (Klinghammer and Goodman, 1987; Udell et al., 2008; Range and Virányi, 2011) and testing (Moretti et al., 2015; Marshall-Pescini et al., 2017, please see Study Animals section in the Methods below). We tested three litters of wolves (*N* = 13) and two litters of dogs (*N* = 12), hand-raised under identical conditions, at 6, 10, 14, 18, 22, and 26 weeks of age (i.e., before sexual maturity) in repeated novel object tests. We used a new novel object in each of the six tests, choosing vastly different objects between tests to avoid the risk of habituation (van Oers et al., 2005; Noer et al., 2015). Novel objects were of different shape, size, color, and texture, and some objects included the element of sound and/or movement, similar to objects that have previously been used in novel object tests on dogs and wolves (Moretti et al., 2015; Marshall-Pescini et al., 2017). The novel object test is an established method to quantify fear and exploration of novelty and has been used on numerous species (Bremner-Harrison et al., 2004; Boogert et al., 2006; Mainwaring et al., 2011; Moretti et al., 2015; Marshall-Pescini et al., 2017). As is commonly applied in novel object tests, we used latency to approach the novel object to quantify fear (Boissy, 1995; Malmkvist and Hansen, 2002; Meehan and Mench, 2002; Ley et al., 2007; Moretti et al., 2015). Our longitudinal design allowed us to assess fear development and expression in juvenile wolves and dogs over an unprecedented period of time, and address our overall goal to test the hypothesis that domestication has altered fear responses in dogs compared to wolves. Based on studies reporting delayed onset of fear behavior in domestic species (Belyaev et al., 1985; Coppinger and Coppinger, 2001; Martin and Fitzgerald, 2005; Lord, 2013), we expected wolves to express exaggerated fearfulness compared to dogs already at 6 to 10 weeks of age by increasing their latency to approach the novel object.

## Materials and Methods

### Ethical Statement

Daily care and all experiments were performed in accordance with relevant guidelines and regulations under national Swedish Law. The experimental protocols in this study were approved by the Ethical Committee in Uppsala, Sweden (approval number: C72/14). Facilities and daily care routines were approved by the Swedish National Board of Agriculture (approval number: 5.2.18-12309/13).

### Study Animals

Between 2014 and 2016, two litters of Alaskan huskies (*N* = 12) and three litters of European gray wolves (*N* = 13) were hand-raised and extensively socialized under similar conditions from the age of 10 days. This set-up was chosen to minimize environmental bias, including maternal effects, which is well-documented to affect the development of behavioral patterns (Clark and Galef, 1982; Wilsson and Sundgren, 1998; Bray et al., 2017). The Alaskan husky is a not a registered dog breed, but a type of dog specifically bred for dog sledding, consisting of a blend of registered dog breeds including Greenland Dog, Siberian Husky, Alaskan Malamute and various pointer breeds. Besides the issue of availability, Alaskan husky was our dog type of choice based on the morphological similarities with wolves (i.e., erect ears, similar size, long snouts etc.). This study was part of a bigger project to investigate domestication-driven changes in behavioral ontogeny in dogs and wolves, including social behavior such as dominance. Thus, it was important to ensure that wolves and dogs in the project had the same morphological basis providing them with equal opportunities to perform the same behavioral repertoires. The dog litter from 2014 consisted of five males and one female and the 2015 litter of three males and three females. The three wolf litters consisted of three females and two males in 2014, two males in 2015 and four males and two females in 2016. The wolf litters from 2014 and 2015 were full siblings and not related to the wolf litter from 2016. The dog litters were unrelated.

Puppies (both dogs and wolves) were raised within litters and socialization involved 24-hour presence of human caregivers for the first 2 months. From 2 months of age, caregiver presence was decreased with a few hours a day until 3 months of age and then further decreased during every other night at 4 months of age. At 6 months of age, caregivers spent 4–6 h with the puppies a day. All wolf and dog litters were kept separate, but reared under standardized conditions. From the age of 10 days to 5 weeks, puppies were reared in identical indoor rooms and here after given access to smaller roofed outdoor enclosures. After a week of habituation to the roofed outdoor enclosure, puppies were given access to a larger fenced grass enclosure at 6 weeks of age. Hereafter the puppies had free access to all three enclosures during the day and access to the indoor room and the roofed enclosure during the night. When the puppies where 3 months old they were moved to large outdoor enclosures (2,000 square meters), in which they remained for the rest of the study period. We started behavioral observations at 10 days of age and behavioral testing was initiated at 6 weeks of age. Testing procedures and exposure to the new environments were standardized over the 3 years. As required by national law, all hand-raisers were ethically certified and trained to handle animals. Furthermore, rules were implemented to assure that rearing was standardized across all caregivers. This included that puppies were never disciplined or trained, and that puppies had access to the same enrichment (starting at 2 weeks of age) and exposure to the environment at the field station, which included strangers passing by the enclosure, vehicles etc. (from 5 weeks of age).

### Experimental Design

To investigate the ontogeny of fear expression in wolves and dogs, we designed a longitudinal experiment with novel object testing once a month starting at 6 weeks of age and ending at 26 weeks of age. The reason we chose to start testing at 6 weeks of age was to ensure that the puppies' senses were fully developed (Lord, 2013). Novel object tests were hereafter performed on a monthly basis at 10, 14, 18, 22, and 26 weeks of age using protocols similar to previous studies subjecting wolves and dogs to novel object tests (Moretti et al., 2015; Marshall-Pescini et al., 2017). To avoid environmental bias and disturbances by testing wolves and dogs in their outdoor home enclosures, we chose to conduct our tests in an indoor testing arena, which was familiar to both wolves and dogs. The equal familiarity among wolves and dogs with the test room also ensured that animals would focus on the novel object and not a novel environment (Moretti et al., 2015). A novel object was presented in the test room (5 × 5 meters), placed opposite of where the puppy would enter the room, approximately four meters away from the door. This placement of the novel object ensured that puppies would actively have to approach the object to investigate and interact with it. Puppies were led into the room by a caregiver, who quickly left the room and closed the door. The duration of a trial was 10 min and trials were always monitored. Eleven trails (all wolves) were stopped prematurely. An interesting observation in this regard was that five of these cases occurred in the test at 26 weeks. In this test one male and one female wolf from 2014, one male wolf from 2015 and two male wolves from 2016 all chewed over the line holding the moving sheet suspended from the ceiling. In all cases this took place after the wolves had observed the moving sheet for a short while and then pulled down the sheet to bite the line, or jumped straight for the line. The other tests stopped prematurely were all in week 18 and 22. These tests were stopped to avoid that the wolves destroyed the novel object. All tests were filmed with two mounted GoPro cameras (model 3-4, GoPro Inc.) on opposite sides of the room (see Videos S1, S2).

### Novel Objects

Due to the repeated exposure to novel objects in our experimental design, we chose vastly different objects between tests to avoid the risk of habituation (van Oers et al., 2005; Noer et al., 2015). We chose novel objects of different shape, size, color and texture, similar to objects that have previously been used for novel object tests on dogs and wolves (Moretti et al., 2015). Increasing the complexity of the novel object, such as adding sound or movement, has previously been used to avoid maturity and/or experience effects on habituation in novel object tests (Malmkvist et al., 2012). Thus, as a way of implementing complexity in later tests (week 22 and 26) we added movement and/or sound to the novel object, i.e., a mechanical dog and a moving bed sheet, respectively. Moving objects are well known to elicit fear responses (Boissy, 1995) and mechanical toys have previously been used in novel object tests on wolves and/or dogs (Plutchik, 1971; Goddard and Beilharz, 1984; King et al., 2003; Marshall-Pescini et al., 2017). As we wished to test the response toward a fear eliciting stimuli in general, including social fear (Gray, 1987), we opted to use a mirror as a novel object in week 14. While mirrors have previously been used in novel object tests to mimic a novel social context (Noer et al., 2015), we acknowledge that the use of a mirror to quantify fear responses might be considered controversial, and we therefore analyzed our data both with and without the test at week 14 (see Statistical methods below).

According to procedures in previous novel object tests on wolves and dogs (Moretti et al., 2015), objects were handled as little as possible and always with freshly washed hands to avoid food smells transferring to the objects and possibly affecting the puppy's behavior toward the object. Novel objects chosen at 6 weeks were: a rolled up mattress, 10 weeks: a wheelbarrow (up-side down), 14 weeks: a mirror mounted to the wall, 18 weeks: a stuffed wolverine toy, 22 weeks: a moving mechanical dog and a moving bed sheet (attached to a string) at 24 weeks.

### Behavioral Scoring

We chose our behavioral categories based on basic behaviors directed at the novel object and behaviors not directed at the novel object (Table 1a). Besides using latency to approach as our measurement of fear, we also included other behaviors previously used in novel object tests for dogs and wolves (Moretti et al., 2015; Marshall-Pescini et al., 2017), such as interaction with the novel object, for further interpretation of our results. Behaviors in this section of the ethogram were scored with clear, non-overlapping segregation with prioritization of behaviors directed at the novel object. For instance, if the puppy was looking at the novel object while moving around the test room this was scored as *looking at novel object* and not *active behavior*. We also attempted to graduate the behaviors directed at the novel object based on the puppies' distance from the novel object. For example, we differentiated between the categories of *investigating novel object* and *looking at novel object*, based on how close the puppy was to the novel object (Table 1a). Behaviors were classified as durations, i.e., seconds (Tables S1–S3). Similar to previous studies (Moretti et al., 2015), *latency to approach the novel object* was measured as the duration from test start to the time the puppy came within a distance of 1 m from the novel object, and *latency to make contact with the novel object* was measured as the time lag to make physical contact with the novel object for the first time *after* the novel object had been approached within a distance of 1 meter.

**Table 1:** Ethogram.

**Behavior**	**Description**
**a) Basic behaviors**	
Active behavior	Moving around in, or interacting with, the test room with no attention to the novel object
Investigating novel object	Sniffing novel object or looking novel object form <1 meter
Latency to approach novel object	Time delay to approach the novel object with <1 meter
Latency make contact with novel object	Time delay to physically touch the novel object (sniffing) after having approached the object within a distance of <1 meter
Looking at novel object	Looking at novel object from a distance of more than 1 meter
Manipulating novel object	Pawing, nosing, scratching, biting, carrying, standing on novel object
Passive behavior	Standing, sitting or lying passively with no attention to the novel object or the test room, including by the door
**b) Fear behaviors**	
Fleeing	Turning from the object in a sudden movement and running away
Growl	Low guttural sound in the throat
Lowered body posture	Head, front, or entire body is lowered, possibly crouching
Piloerection	Hairs on neck and/or back are raised
Retreat	The approach to the novel object is halted and the puppy backs up
Startle	Sudden, short jolt of head or entire body
Tugged tail	Tugging tail between hind legs, possibly all the way up to the stomach

*Behaviors scored during novel object tests. Basic behaviors (a) were scored in a non-overlapping way, with prioritization of behaviors related to the novel object. Latency times were measured regardless of the behavior performed. Fear behaviors (b) were scored only when the puppy had its focus on the novel object, i.e., during looking at novel object, investigating novel object, and approaches. Fear behaviors were scored in an overlapping manner*.

Avoidance behavior and latency to approach a novel object are commonly applied to quantify fearfulness in various animal species (Boissy, 1995; Malmkvist and Hansen, 2002; Meehan and Mench, 2002), including dogs and wolves (Ley et al., 2007; Moretti et al., 2015). However, to confirm that that a longer latency to approach the novel object was related to fear and not disinterest in our study, we also assessed fear behaviors across our tests (Table 1b, Table S4). Differences in body posture are sometimes used as an indication of fear expression in both wolves and dogs (King et al., 2003; Stellato et al., 2017; Rao et al., 2018). Yet, dogs can express altered body posture in neutral test conditions, i.e., when no novel object is present (Stellato et al., 2017). Though dogs and wolves in our study were tested in a familiar room, we cannot rule out that confinement in an isolated room did not affect individuals differently. Therefore, to avoid potential bias in assessing body postures, and other behaviors related to fear, the behaviors in the fear part of the ethogram were only scored when the puppy was focused on the novel object (i.e., looking at it, approaching it etc.). We noted that fear behaviors were not expressed toward elements other than the novel object. Wolves and dogs expressed similar repertoires of fear behaviors (Table S4) and behavioral scoring of fear behavior included the entire duration of a trial for all puppies. Some fear behaviors would overlap, such as tugged tail and growling or piloerection and lowered body posture, and were scored as such. No puppy showed fear behavior after the initial investigation of the novel object. Puppies not approaching or making contact with the novel object continued to express fear behaviors throughout the duration of the test. As reported in other studies quantifying fear in dogs using novel objects (Stellato et al., 2017), the occurrence of subtle behaviors such as auto-grooming, barking, tail wagging and yawning was limited and we therefore chose to not include these behaviors in our analyses.

Behavioral scoring was carried out using the software BORIS v. 5.1.3 (Friard and Gamba, 2016). Based on cross coding, reliability of the behavioral scoring was calculated using Cohen's kappa and was considered good with a value of 87.4%.

### Statistical Methods

We tested for the effect of species in each behavior by using a mixed model strategy with the fixed effects of interest being species, age, their interaction and sex. Additionally, we adjusted our models for the effect of differences in trial duration by including duration as a covariate in our models (except for latency models), and by adding the durations as weights. To account for the repeated measures of individuals and the non-independence of individuals with shared genetic variation, we included random intercepts for both factors. The full model in lme4 syntax: y ~ species ^*^ age + sex + duration + (1|individual) + (1|relatedness) (see Table S5 for random effects estimates). We centered the age variable to aid interpretation of the species effect in the presence of the interaction. Models were compared to a null model using AIC (cut-off ΔAIC > 2, Burnham and Anderson, 2004, Table S6) to check whether to remove the interaction between species and age.

To model latencies, we used survival analysis through mixed effects Cox models. All but four puppies (dogs: *N* = 2, wolves: *N* = 2, Table S1) approached the novel object within a distance of 1 meter, and we assigned the total test time as latency to approach for the four puppies that did not approach the novel object. In eight cases (dogs: *N* = 6, wolves: *N* = 2) puppies did not make contact with the novel object (Table S1). Since we defined the latency to contact as starting after the initial approach, four of these puppies did approach the object and their latencies were coded as right-censored with a value of (trial duration—latency to approach). For the four trials where puppies did not approach or make contact with the object, no information about the latency to contact was available and these were coded as a right-censored latency of 1 s. The time spent looking, investigating and manipulating the novel object were modeled using GAMLSS with a log-normal distribution, in order to fulfill the assumption of normality of the model residuals. We added 1 to these variables, to avoid having undefined values for observations with 0 values. The active and passive behavior variables were modeled using linear mixed models (i.e., using lme4). We visually confirmed normality of residuals for the appropriate models. *P*-values for the lme4 models were obtained using Satterwaithe's approximation of denominator degrees of freedom.

To aid in the interpretation of the development of latency to approach with age, we used the Cox regression to estimate the marginal effects of species at each age point in the experiment. *P*-values were adjusted for multiple testing using Holm's method (Table S8). Additionally, we calculated the estimated marginal means of the age trends for dogs and wolves, to test whether each species significantly showed altered development of the latency to approach with age (Table S9).

To rule out that using a mirror as a novel object did not affect our results, we re-ran all our analyses without the test at week 14 (Tables S10–S12). We found that our results were similar in analyses excluding and including the mirror, and we present the analyses including the test at week 14 below. Lastly, to rule out that manipulation of the novel object was not affected by object type, we also performed as separate analyses for the time spent manipulating the novel object using only tests in which the novel object was more inviting for manipulation. To do this we performed the identical analyses as described above only on the trials at week 18, 22, and 26 (Tables S13–S15, Figure S1).

All statistical analyses were performed in R (v3.4.3, R Core Team 2016), with mixed effects models fitted using the package *lme4* v. 1.1–15 (Bates et al., 2015), survival analysis using *coxme* (Therneau, 2018), Satterwaithe's approximation from *lmerTest* v. 2.0-36 (Kuznetsova et al., 2017), GAMLSS using *gamlss* (Rigby and Stasinopoulos, 2005), and marginal means were estimated using *emmeans* (Lenth, 2019).

## Results

### Latency Measures

The number of fear behaviors expressed in relation to the novel object (Table S4) was positively correlated with the latency to approach across trials (Spearman Rank ρ = 0.188, *p* = 0.023), thereby confirming that increased latency to approach is an expression of fear, and not disinterest in the novel object.

**Figure 1:**
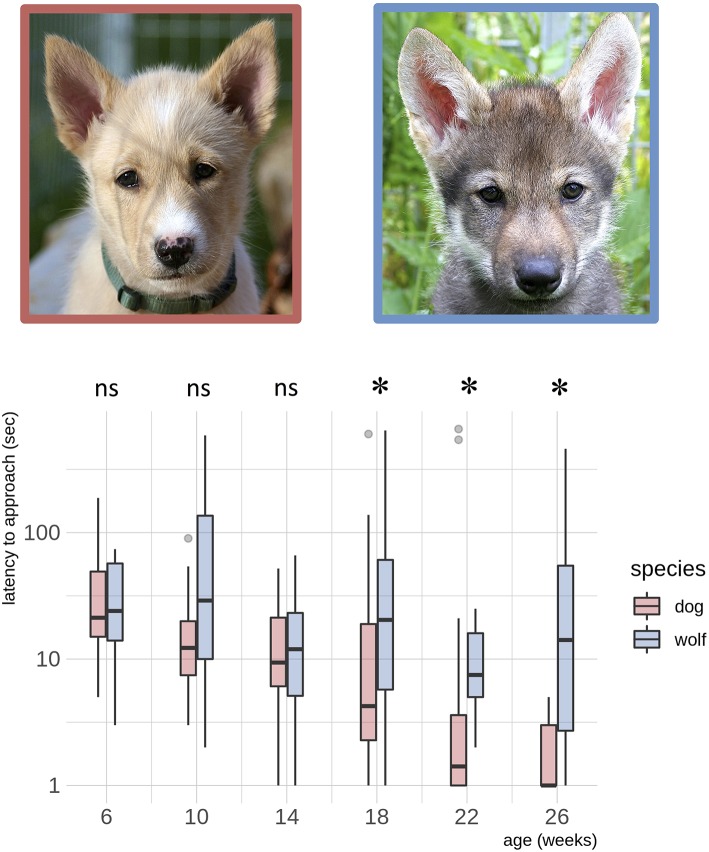
Dog – wolf comparisons, latency to approach. Boxplots show behavioral scores during a novel object test, comparing dogs and wolves across age. Overlaid are the fits and confidence intervals from the best model, selected by AIC. Boxes indicate the quartiles, and the whiskers reach maximally 1.5 times the interquartile range. Values beyond that are shown as points. A log(y) scale) was used. Species differences in latency to approach the novel object are significant from the age of 18 weeks (indicated by ^*^) (Table S7). Photos: Christina Hansen Wheat.

Wolves and dogs developed differently in latency to approach the novel object within 1 meter (*z* = −2.23, *p* = 0.026, Table 2, Figure 1). At the age of 18 weeks, dogs approached the novel object significantly faster than wolves (*z* = 2.51, *p*_adjusted_ = 0.048, Figure 1, Table S8) and this difference was maintained at 22 weeks (*z* = 2.92, *p*_adjusted_ = 0.018, Figure 1, Table S8) and 26 weeks (*z* = 2.97, *p*_adjusted_ = 0.018, Figure 1, Table S8). This species difference was driven by dogs significantly decreasing their latency to approach with age (slope estimate [95%CI]: 0.1065 [0.054, 0.159], Table S9), whereas wolves maintained similar latencies to approach with age (slope estimate [95%CI]: 0.0192 [-0.007, 0.045], Table S9).

**Table 2:** Model summary.

**Behavior**	**Term**	**Est**.	**Std. error**	**z**	**t**	***p***
Latency, approach	specieswolf	−0.476	0.23	−2.07		***0.038***
	age_centered	0.087	0.022	4		*** <0.001***
	sexMale	0.345	0.242	1.43		0.15
	specieswolf:age_centered	−0.062	0.028	−2.23		***0.026***
Latency, contact	specieswolf	−0.202	0.174	−1.16		0.24
	age_centered	−0.059	0.019	−3.08		***0.002***
	sexMale	−0.05	0.179	−0.28		0.78
	specieswolf:age_centered	0.041	0.025	1.66		0.096
Looking at NO	(Intercept)	−0.533	1.313		−0.406	0.686
	specieswolf	0.055	0.329		0.166	0.884
	age_centered	0.121	0.021		5.798	*** <0.001***
	sexMale	0.356	0.337		1.055	0.304
	duration	0.032	0.124		0.259	0.796
	specieswolf:age_centered	−0.065	0.032		−2.054	***0.042***
Investigating NO	(Intercept)	−2.725	1.142		−2.386	***0.019***
	specieswolf	0.126	0.189		0.667	0.573
	age_centered	−0.12	0.019		−6.425	*** <0.001***
	sexMale	−0.1	0.19		−0.529	0.603
	duration	0.268	0.11		2.439	***0.016***
	specieswolf:age_centered	0.055	0.028		1.93	*0.056*
Manipulating NO	(Intercept)	1.032	1.901		0.543	0.588
	specieswolf	0.491	0.346		1.419	0.292
	age_centered	−0.063	0.031		−2.032	***0.044***
	sexMale	−0.489	0.318		−1.538	0.14
	duration	−0.097	0.183		−0.53	0.597
	specieswolf:age_centered	0.066	0.047		1.406	0.162
Active behavior	(Intercept)	−95.725	97.292		−0.984	0.327
	specieswolf	101.479	21.934		4.627	*** <0.001***
	age_centered	2.682	1.558		1.721	0.088
	sexMale	−8.563	22.387		−0.383	0.706
	duration	31.552	9.247		3.412	***0.001***
	specieswolf:age_centered	−0.318	2.374		−0.134	0.894
Passive behavior	(Intercept)	134.169	85.69		1.566	0.124
	specieswolf	−81.338	50.903		−1.598	0.251
	age_centered	−5.153	1.276		−4.037	*** <0.001***
	sexMale	4.929	16.245		0.303	0.765
	duration	10.437	7.547		1.383	0.169
	specieswolf:age_centered	2.444	1.94		1.259	0.21

*Results for the best fitted model of repeated measures, with dogs as the reference, on (1) Latency to approach the novel object, (2) Latency to make contact with the novel object, (3) Looking at novel object (NO), (4) Investigating novel object, (5) Manipulating novel object, (6) Active behavior, and (7) Passive behavior. Estimate, standard error, test statistic (z or t) and p-values are given. Significant p-values are marked in bold italic*.

**Figure 2:**
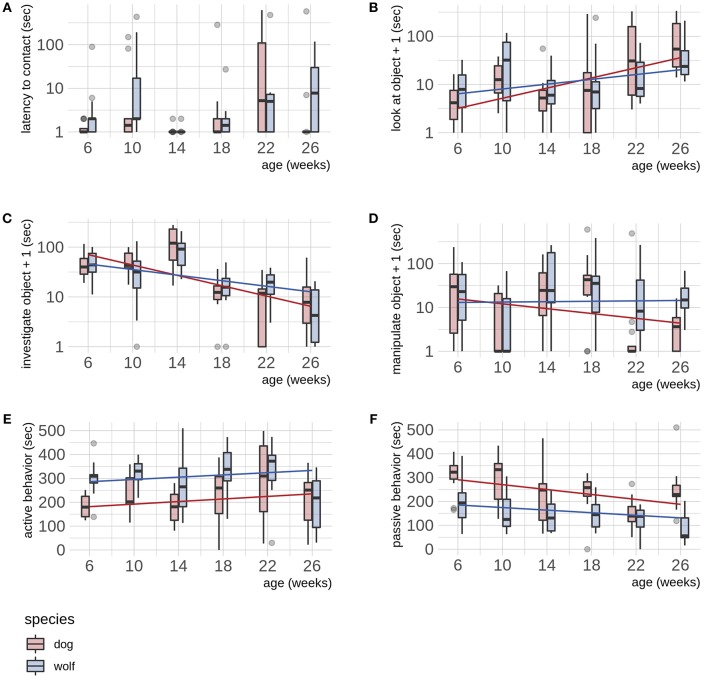
**(A–F)** Dog—wolf comparisons. Boxplots show behavioral scores during a novel object test, comparing dogs and wolves across age. Overlaid are the fits and confidence intervals from the best model, selected by AIC. Boxes indicate the quartiles, and the whiskers reach maximally 1.5 times the interquartile range. Values beyond that are shown as points. Note that b makes use of a log(y) scale, and panels d, e and f use log(y + 1). Note that the interaction term is only significant for **(B)** (see Table 2).

For the latency to make contact with the novel object, we found no differences in wolves and dogs (Table 2, Figure 2A). We did not find evidence of sex differences in either species in either latency measurements.

### Behaviors Related to the Novel Object

We found that wolves and dogs developed differently in looking at the novel object (*t* = −2.054, *p* = 0.042, Table 1, Figure 2B), although *post hoc* testing did not reveal significant differences at any age (Table S8). Both wolves and dogs increased their time looking at the novel object with age (*F* = 33.99, *p* ≤ 0.001, Table S7). Wolves and dogs showed similar developmental trajectories for the time spent investigating the novel object (Table 2, Figure 2C), with an overall decrease with age in both species (*F* = 56.78, *p* ≤ 0.001, Table S7). Wolves and dogs also developed similarly in time spent manipulating the novel object (Table 2, Figure 2D). The analyses for manipulation of the novel object only for the objects considered more inviting for manipulation (i.e., week 18, 22, and 26) were qualitatively similar to the main analyses and revealed no species differences or effect of age (Tables S13–S15, Figure S1). There was no evidence of sex differences in either species in either of the behaviors related to the novel object.

### Behaviors Not Related to the Novel Object

We found that wolves expressed higher levels of activity than dogs throughout the test period (*t* = 4.627, *p* ≤ 0.001, Table 2, Figure 2E) and that both species increased their activity with age (*F* = 4.47, *p* = 0.036, Table S7). Passive behavior decreased with age in both wolves and dogs (*F* = 16.25, *p* ≤ 0.001, Figure 2F, Table S6), and while dogs appeared more passive than wolves, the species differences was not significant. We found no evidence of sex differences in either species in behaviors not related to the novel object.

## Discussion

Decreased expression of fear is considered a key behavioral alteration in domesticated animals, and it has further been suggested that domestication drives altered developmental rates delaying the initial onset of fear response (Belyaev et al., 1985). However, few studies have actually tested this experimentally and for wolves and dogs specifically, it remains unclear if and how a developmental shift during early ontogeny affects the continued development and expression of fear in either species. Here we present the first extended examination of the development of fear behavior within the juvenile period in wolves and dogs. Contrary to expectations, we found no evidence in support of an increase in fearfulness in wolves with age or a delayed onset of fear response in dogs. Instead we found that dogs significantly reduced their fear response to a novel object in the period between 6 and 26 weeks of age. Critically, we did not detect differences in wolves' fear response toward novelty with age, and the detected species difference can be attributed to a progressively reduced fear response in dogs. Together our results suggest that species differences in fear of novelty between wolves and dogs are not caused by a domestication-driven shift in the first onset of fear response. Instead, we suggest that a loss of sensitivity toward novelty with age in dogs causes the difference in fear expression toward novelty in wolves and dogs.

We formally tested the general expectation that domestication has caused a delay in the sensitive period in dogs, resulting in later onset of fear behavior compared to wolves (Scott and Fuller, 1965; Fox, 1970; Zimen, 1987; Coppinger and Coppinger, 2001; Lord, 2013) by quantifying latency to approach novel objects in wolves and dogs. While we predicted differences in fear expression in wolves and dogs already at 6 or 10 weeks of age, with wolves expressing exaggerated fear responses to novelty, we detected no such species differences during early development. This finding is in agreement with a recent study comparing exploration of novelty in 6 and 8 weeks old wolves and dogs, which showed that wolves and dogs did not differ in their expression of fear behaviors or the latency to make contact with a novel object (Marshall-Pescini et al., 2017). Yet, adult wolves express increased latency to make contact to a novel object compared to dogs (Moretti et al., 2015), thereby suggesting that species differences in fear expression might arise later in development than previously thought. However, the lack of extended, temporal studies on fear development has so far left this issue unresolved. Our finding that a species difference in latency to approach a novel object occurred from 18 weeks of age and onwards thereby represents the first indication of when a quantifiable difference in fear toward novelty arises in wolves and dogs. Importantly, this species difference did not occur because wolves became more fearful with age, as expected, but rather because dogs decreased their time to approach the novel object, which suggests that dogs, but not wolves lose their sensitivity toward novelty with age.

Upon subjecting individuals to repeated novel object tests, and although objects differ between trials, there is a risk of habituation to novelty itself (Réale et al., 2007), and such a generalization of novelty *per se* can affect the potential to interpret fear responses from novel object tests. However, in showing a positive relationship between latency to approach the novel object and the number of fear behaviors expressed, we were able to rule out that disinterest in the novel object or habituation to the test situation, and not fear, were driving long latencies to approach in our study. Fear of novelty was expressed immediately in both wolves and dogs through a delayed latency to approach and once the novel object was approached this initial fearfulness appeared to no longer affect behavioral responses in either species. This is reflected in the lack of species differences in latency to make contact with, investigate or manipulate the novel object, and the fact that fear behaviors were not observed in any individual after initial contact with the novel object had been made. The equal interest between wolves and dogs in interacting with the novel object contrasts with previous findings that both juvenile and adult wolves show increased interest in investigating and manipulating novel objects, while dogs seem to lose interest in interacting with novel objects with age (Moretti et al., 2015; Marshall-Pescini et al., 2017). In our study, behaviors that are more closely related to the novel object itself, i.e., latency to contact, looking at, investigating and manipulating the novel object, show more variability across tests than latency to approach and behaviors not related to the novel object. This variability was most likely caused by the different novel objects used and it is possible that the increased variance may have prohibited detection of additional species differences in behavioral measures directly related to the novel object. Importantly, the development in latency to approach the novel object in both wolves and dogs appeared to be less affected by the choice of novel object, indicating that latency to approach was more influenced by novelty itself.

Different paces in physical developmental in wolves and dogs could potentially influence our results. First, wolves develop physically faster than dogs (Frank and Frank, 1982), and it has been suggested that wolves express increased activity at an earlier age than dogs due to this difference in developmental pace of motor patterns (Frank and Frank, 1982; Marshall-Pescini et al., 2017). However, while we do find a species difference in how much time is spent on active behavior during tests, this species difference is consistent across age and not restricted to early ontogeny alone. This indicates that wolves, on a general scale, are more active when in the test room than dogs. While it cannot be ruled out that active behavior is affected by the presence of a novel object, it is a less likely explanation for our findings as we measured behaviors in a non-overlapping way with priority of behaviors related to the novel object. Thus, the measurement of activity does not include looking at, manipulating or approaching the novel object, but only time spent on active behavior with no attention to the novel object. Instead the higher activity in wolves might reflect an increased reactivity of being separated from littermates and being confined in the test room compared to dogs. Second, earlier sexual maturity in dogs compared to wolves (Morey, 1994; Goodwin et al., 1997) might explain the rapid decline in fearfulness in dogs, but not wolves, in our study. However, captive wolves removed from social constraints of pack-living, and thus potentially behavioral suppression of reproductive development, sexually mature as early as 9 months of age (Medjo and Mech, 1976), which is comparable to sexual maturation in dogs (Morey, 1994). As our study compared wolves and dogs living in captive, non-reproductive groups before the occurrence of sexual maturity, and as we found no effect of sex on the expression of behavior in either species, we find it unlikely that differences in sexual development are driving, nor are relevant, for our results.

Here we have compared behavioral development in wolves and dogs using standardized methods in both hand-raising, socialization (Klinghammer and Goodman, 1987; Udell et al., 2008; Range and Virányi, 2011) and testing (Moretti et al., 2015; Marshall-Pescini et al., 2017), thereby making our study comparable to some of the previous findings on fear development in the two species. Subsequently, our reporting of previously undetected variation in the development of fear expression is highly relevant for the on-going discussion of behavioral implications of domestication in dogs. In conclusion, our study shows that wolves and dogs do not differ in their fear toward novelty from 18 weeks of age and onwards because dogs, but not wolves, become less fearful with age. We acknowledge that, as in other studies comparing hand-raised wolves and dogs, our results are limited by small sample sizes and we note that although we found significant support for a difference in fear development between dogs and wolves, the individual variation among individuals creates an uncertainty in the magnitude of the effects found. Furthermore, various dog breeds such as Poodle (Feddersen-Petersen, 1991), Alaskan Malamute (Frank and Frank, 1985) and German Shepherd, Siberian Husky, Alaskan Malamute, Czechoslovakian Wolfdog (Hansen Wheat et al., 2018) as well as mixed breeds (Range et al., 2015; Marshall-Pescini et al., 2017) have been used to uncover the behavioral implications of domestication from wolves. However, with dogs being bred to fulfill highly specialized behavioral niches (Coppinger and Coppinger, 2001; Svartberg, 2006; Mehrkam and Wynne, 2014), results will inevitably vary across studies (Scott and Fuller, 1965; Morrow et al., 2015). Here we have used Alaskan huskies, a mixed breed or “dog type” that due to its heritage arguably can be categorized as an ancient breed (Lindblad-Toh et al., 2005; vonHoldt et al., 2010). Nonetheless, even if our dogs represent a more ancestral stage of dog domestication, it is noteworthy that we do in fact detect a behavioral difference between wolves and dogs in our study. Detection of differences between wolves and dogs, no matter the breed of dog or subspecies of wolf, is of great importance to the continued discussion of the paradigm of domestication-driven changes in behavior. In conclusion, because of the small sample sizes inherently available in studies comparing behavior in wolves and dogs, it is critical that continued, standardized studies on wolf dog comparisons are encouraged to further uncover the resolution in behavioral variation during domestication.

## Data Availability

All datasets generated for this study are included in the manuscript and/or the Supplementary Files.

## Author Contributions

CH and HT designed the study. CH conducted the experiments and prepared data for analyses. WvdB and CH planned how to analyse the data and WvdB analyzed the data. CH wrote the manuscript with input from HT and WvdB. All authors reviewed the manuscript prior to submission.

### Conflict of Interest Statement

The authors declare that the research was conducted in the absence of any commercial or financial relationships that could be construed as a potential conflict of interest.

## Supplementary Material

Video S2 Available online at: https://figshare.com/s/c8e7ea7498c763004138.
