# Supplementary material for: Dogs, but Not Wolves, Lose Their Sensitivity Toward Novelty With Age
Source: Front Psychol. 2019 Sep 4;10:2001. doi: 10.3389/fpsyg.2019.02001 (PMC6742907; doi:10.3389/fpsyg.2019.02001)

Dogs, but not wolves, lose their sensitivity towards novelty with age

Christina Hansen Wheat, Wouter van der Bijl & Hans Temrin

Department of Zoology, Stockholm University, SE-10691 Stockholm, Sweden

Supplemental materials

Table S1. Behavioural scores, latencies………………………………………. 2

Table S2. Behavioural scores, novel object related………………………… 6

Table S3. Behavioural scores, non-novel object related……………………. 10

Table S4. Fear scores………………………………………………………….. 14

Table S5. Random effects estimates………………………………………… 18

Table S6. Model selection……………………………………………………… 19

Table S7. Anova table…………………………………………………………. 20

Table S8. Post hoc testing……………………………………………………. 21

Table S9. Age trends………………………………………………………… 22

Table S10. Model selection, excluding week 14……………………………. 23

Table S11. Model summary, excluding week 14……………………………… 24

Table S12. Anova table, excluding week 14……………………………….. 25

Table S13. Model selection, NO manipulation week 18, 22 and 26……… 26

Table S14. Model summary, NO manipulation week 18, 22 and 26…….. 27

Table S15. Anova table, NO manipulation week 18, 22 and 26………….. 28

Figure S1. NO manipulation week 18, 22 and 26………………………….. 29

Table S1. Behavioural scores, latencies. Latencies for approaching and making contact with the novel object and total test time for each of the six novel object tests. Latency to approach (Lat_app) is measured as the time in seconds from test start to the puppy comes within a 1 meter radius of the novel object. Latency to make contact (Lat_con) is measured as the time lag in seconds to make physical contact with the novel object after it has been approached. Values in parentheses are right-censored (see methods).

Individual	Sex	Species	Litter	Novel object	Lat_app	Lat_con	Total time
Bowie_6w	M	dog	2014	Rolled up mattress	5	1	600.076
Bowie_10w	M	dog	2014	Wheelbarrow	90	1	587.139
Bowie_14w	M	dog	2014	Mirror	35	1	612.262
Bowie_18w	M	dog	2014	Stuffed wolverine	26	1	609.377
Bowie_22w	M	dog	2014	Mechanical dog	1	1	603.233
Bowie_26w	M	dog	2014	Moving sheet	1	(575.78)	576.78
Cash_6w	M	dog	2014	Rolled up mattress	46	2	601.049
Cash_10w	M	dog	2014	Wheelbarrow	54	1	627.571
Cash_14w	M	dog	2014	Mirror	12	1	574.068
Cash_18w	M	dog	2014	Stuffed wolverine	1	1	614.802
Cash_22w	M	dog	2014	Mechanical dog	1	301	607.46
Cash_26w	M	dog	2014	Moving sheet	1	1	609.681
Jagger_6w	M	dog	2014	Rolled up mattress	188	1	603.606
Jagger_10w	M	dog	2014	Wheelbarrow	32	1	604.013
Jagger_14w	M	dog	2014	Mirror	52	1	609.974
Jagger_18w	M	dog	2014	Stuffed wolverine	1	1	605.033
Jagger_22w	M	dog	2014	Mechanical dog	1	(616.042)	617.042
Jagger_26w	M	dog	2014	Moving sheet	1	1	605.032
Janis_6w	F	dog	2014	Rolled up mattress	60	1	604.807
Janis_10w	F	dog	2014	Wheelbarrow	10	1	608.57
Janis_14w	F	dog	2014	Mirror	4	1	622.112
Janis_18w	F	dog	2014	Stuffed wolverine	138	2	606.243
Janis_22w	F	dog	2014	Mechanical dog	(658.976)	(1)	658.976
Janis_26w	F	dog	2014	Moving sheet	1	1	594.822
Lennon_6w	M	dog	2014	Rolled up mattress	82	2	606.69
Lennon_10w	M	dog	2014	Wheelbarrow	15	149	610.654
Lennon_14w	M	dog	2014	Mirror	22	1	601.694
Lennon_18w	M	dog	2014	Stuffed wolverine	1	1	616.13
Lennon_22w	M	dog	2014	Mechanical dog	1	9	606.609
Lennon_26w	M	dog	2014	Moving sheet	1	1	594.675
Marley_6w	M	dog	2014	Rolled up mattress	30	1	604.525
Marley_10w	M	dog	2014	Wheelbarrow	3	1	623.797
Marley_14w	M	dog	2014	Mirror	3	1	613.112
Marley_18w	M	dog	2014	Stuffed wolverine	(601.682)	(1)	601.682
Marley_22w	M	dog	2014	Mechanical dog	1	(605.481)	606.481
Marley_26w	M	dog	2014	Moving sheet	1	1	603.068

Table S1, continued

Individual	Sex	Species	Litter	Novel object	Lat_app	Lat_con	Total time
Björk_6w	F	wolf	2014	Rolled up mattress	36	1	579.774
Björk_10w	F	wolf	2014	Wheelbarrow	3	1	595.852
Björk_14w	F	wolf	2014	Mirror	1	1	631.068
Björk_18w	F	wolf	2014	Stuffed wolverine	5	1	616.321
Björk_22w	F	wolf	2014	Mechanical dog	2	2	598.221
Björk_26w	F	wolf	2014	Moving sheet	60	1	609.076
Iggy_6w	M	wolf	2014	Rolled up mattress	6	2	600.857
Iggy_10w	M	wolf	2014	Wheelbarrow	28	1	610.047
Iggy_14w	M	wolf	2014	Mirror	13	1	615.548
Iggy_18w	M	wolf	2014	Stuffed wolverine	5	2	623.099
Iggy_22w	M	wolf	2014	Mechanical dog	16	477	638.666
Iggy_26w	M	wolf	2014	Moving sheet	1	28	414.633
Joni_6w	F	wolf	2014	Rolled up mattress	6	2	573.469
Joni_10w	F	wolf	2014	Wheelbarrow	236	3	632.978
Joni_14w	F	wolf	2014	Mirror	11	1	649.435
Joni_18w	F	wolf	2014	Stuffed wolverine	70	2	587.12
Joni_22w	F	wolf	2014	Mechanical dog	5	5	609.673
Joni_26w	F	wolf	2014	Moving sheet	460	68	612.939
Lita_6w	F	wolf	2014	Rolled up mattress	14	1	616.709
Lita_10w	F	wolf	2014	Wheelbarrow	5	2	623.776
Lita_14w	F	wolf	2014	Mirror	66	1	617.91
Lita_18w	F	wolf	2014	Stuffed wolverine	16	2	624.842
Lita_22w	F	wolf	2014	Mechanical dog	7	5	593.112
Lita_26w	F	wolf	2014	Moving sheet	2	1	238.123
Ozzy_6w	M	wolf	2014	Rolled up mattress	24	1	589.697
Ozzy_10w	M	wolf	2014	Wheelbarrow	10	2	619.074
Ozzy_14w	M	wolf	2014	Mirror	11	1	626.846
Ozzy_18w	M	wolf	2014	Stuffed wolverine	8	1	596.166
Ozzy_22w	M	wolf	2014	Mechanical dog	25	8	256.226
Ozzy_26w	M	wolf	2014	Moving sheet	4	36	630.475
Billie_6w	F	dog	2015	Rolled up mattress	16	1	618.307
Billie_10w	F	dog	2015	Wheelbarrow	17	2	606.824
Billie_14w	F	dog	2015	Mirror	8	2	622.631
Billie_18w	F	dog	2015	Stuffed wolverine	13	5	617.803
Billie_22w	F	dog	2015	Mechanical dog	542	(77.881)	619.881
Billie_26w	F	dog	2015	Moving sheet	1	1	624.415
Ella_6w	F	dog	2015	Rolled up mattress	15	1	617.097
Ella_10w	F	dog	2015	Wheelbarrow	15	2	608.453
Ella_14w	F	dog	2015	Mirror	1	1	624.353
Ella_18w	F	dog	2015	Stuffed wolverine	17	284	617.032
Ella_22w	F	dog	2015	Mechanical dog	21	19	608.397
Ella_26w	F	dog	2015	Moving sheet	3	1	605.549

Table S1, continued

Individual	Sex	Species	Litter	Novel object	Lat_app	Lat_con	Total time
Muddy_6w	M	dog	2015	Rolled up mattress	15	2	614.377
Muddy_10w	M	dog	2015	Wheelbarrow	9	1	625.693
Muddy_14w	M	dog	2015	Mirror	8	1	619.128
Muddy_18w	M	dog	2015	Stuffed wolverine	3	2	614.359
Muddy_22w	M	dog	2015	Mechanical dog	2	1	622.874
Muddy_26w	M	dog	2015	Moving sheet	3	1	600.184
Red_6w	M	dog	2015	Rolled up mattress	18	1	612.618
Red_10w	M	dog	2015	Wheelbarrow	8	2	631.432
Red_14w	M	dog	2015	Mirror	7	1	618.505
Red_18w	M	dog	2015	Stuffed wolverine	3	1	610.963
Red_22w	M	dog	2015	Mechanical dog	2	3	626.564
Red_26w	M	dog	2015	Moving sheet	1	1	605.679
Simone_6w	F	dog	2015	Rolled up mattress	25	1	621.930
Simone_10w	F	dog	2015	Wheelbarrow	5	2	605.543
Simone_14w	F	dog	2015	Mirror	21	1	609.914
Simone_18w	F	dog	2015	Stuffed wolverine	3	1	624.499
Simone_22w	F	dog	2015	Mechanical dog	1	1	615.770
Simone_26w	F	dog	2015	Moving sheet	5	1	610.149
Skip_6w	M	dog	2015	Rolled up mattress	7	1	610.773
Skip_10w	M	dog	2015	Wheelbarrow	6	81	631.961
Skip_14w	M	dog	2015	Mirror	11	1	621.581
Skip_18w	M	dog	2015	Stuffed wolverine	6	2	614.454
Skip_22w	M	dog	2015	Mechanical dog	2	2	617.542
Skip_26w	M	dog	2015	Moving sheet	3	7	610.066
Flea_6w	M	wolf	2015	Rolled up mattress	3	1	587.411
Flea_10w	M	wolf	2015	Wheelbarrow	172	40	598.813
Flea_14w	M	wolf	2015	Mirror	22	1	609.896
Flea_18w	M	wolf	2015	Stuffed wolverine	108	2	609.496
Flea_22w	M	wolf	2015	Mechanical dog	16	8	602.450
Flea_26w	M	wolf	2015	Moving sheet	25	2	95.161
Hendrix_6w	M	wolf	2015	Rolled up mattress	14	1	593.077
Hendrix_10w	M	wolf	2015	Wheelbarrow	2	2	587.873
Hendrix_14w	M	wolf	2015	Mirror	2	1	612.873
Hendrix_18w	M	wolf	2015	Stuffed wolverine	1	1	623.254
Hendrix_22w	M	wolf	2015	Mechanical dog	8	1	608.376
Hendrix_26w	M	wolf	2015	Moving sheet	2	1	488.468
Elvis_6w	M	wolf	2016	Rolled up mattress	67	2	612.777
Elvis_10w	M	wolf	2016	Wheelbarrow	29	433	626.783
Elvis_14w	M	wolf	2016	Mirror	27	2	637.202
Elvis_18w	M	wolf	2016	Stuffed wolverine	26	1	630.121
Elvis_22w	M	wolf	2016	Mechanical dog	2	1	604.293
Elvis_26w	M	wolf	2016	Moving sheet	54	20	128.490

Table S1, continued

Individual	Sex	Species	Litter	Novel object	Lat_app	Lat_con	Total time
KD_6w	F	wolf	2016	Rolled up mattress	74	5	609.205
KD_10w	F	wolf	2016	Wheelbarrow	136	5	637.961
KD_14w	F	wolf	2016	Mirror	48	1	652.284
KD_18w	F	wolf	2016	Stuffed wolverine	58	27	620.433
KD_22w	F	wolf	2016	Mechanical dog	7	7	613.584
KD_26w	F	wolf	2016	Moving sheet	3	117	481.627
Lemmy_6w	M	wolf	2016	Rolled up mattress	46	2	606.694
Lemmy_10w	M	wolf	2016	Wheel barrel	(586.976)	(1)	586.976
Moby_6w	M	wolf	2016	Rolled up mattress	64	1	617.363
Moby_10w	M	wolf	2016	Wheelbarrow	130	192	627.734
Moby_14w	M	wolf	2016	Mirror	18	1	630.257
Moby_18w	M	wolf	2016	Stuffed wolverine	(641.919)	(1)	641.919
Moby_22w	M	wolf	2016	Mechanical dog	15	5	602.346
Moby_26w	M	wolf	2016	Moving sheet	57	3	106.774
PJ_6w	F	wolf	2016	Rolled up mattress	57	89	580.513
PJ_10w	F	wolf	2016	Wheelbarrow	87	17	615.866
PJ_14w	F	wolf	2016	Mirror	7	1	616.296
PJ_18w	F	wolf	2016	Stuffed wolverine	6	1	603.243
PJ_22w	F	wolf	2016	Mechanical dog	5	1	430.614
PJ_26w	F	wolf	2016	Moving sheet	8	27	468.549
Sting_6w	M	wolf	2016	Rolled up mattress	15	6	608.304
Sting_10w	M	wolf	2016	Wheelbarrow	25	2	616.169
Sting_14w	M	wolf	2016	Mirror	1	1	629.972
Sting_18w	M	wolf	2016	Stuffed wolverine	31	3	616.396
Sting_22w	M	wolf	2016	Mechanical dog	20	1	600.794
Sting_26w	M	wolf	2016	Moving sheet	28	1	194.205

Table S2. Behavioural scores, novel object related. Scores (seconds) for behaviours related to the novel object for each of the six novel object tests.

Individual	Sex	Species	Litter	Novel object	Invest_NO	Look_NO	Manip_NO
Bowie_6w	M	dog	2014	Rolled up mattress	23.011	0	41.449
Bowie_10w	M	dog	2014	Wheelbarrow	39.913	2.298	30.809
Bowie_14w	M	dog	2014	Mirror	55.28	0	0
Bowie_18w	M	dog	2014	Stuffed wolverine	9.95	17.013	45.067
Bowie_22w	M	dog	2014	Mechanical dog	33.833	26.865	3.672
Bowie_26w	M	dog	2014	Moving sheet	0	49.256	0
Cash_6w	M	dog	2014	Rolled up mattress	43.257	3.545	0
Cash_10w	M	dog	2014	Wheelbarrow	29.264	21.581	19.779
Cash_14w	M	dog	2014	Mirror	49.439	1.296	35.033
Cash_18w	M	dog	2014	Stuffed wolverine	7.741	0	44.245
Cash_22w	M	dog	2014	Mechanical dog	25.863	3.595	0
Cash_26w	M	dog	2014	Moving sheet	30.219	25.534	0
Jagger_6w	M	dog	2014	Rolled up mattress	18.154	2.828	46.103
Jagger_10w	M	dog	2014	Wheelbarrow	33.536	17.959	
Jagger_14w	M	dog	2014	Mirror	232.6	5.374	15.608
Jagger_18w	M	dog	2014	Stuffed wolverine	7.937	0	16.449
Jagger_22w	M	dog	2014	Mechanical dog	0	90.557	0
Jagger_26w	M	dog	2014	Moving sheet	5.998	59.871	0
Janis_6w	F	dog	2014	Rolled up mattress	28.373	6.932	2.557
Janis_10w	F	dog	2014	Wheelbarrow	38.738	1.538	0
Janis_14w	F	dog	2014	Mirror	72.683	3.309	8.708
Janis_18w	F	dog	2014	Stuffed wolverine	16.426	36.963	41.729
Janis_22w	F	dog	2014	Mechanical dog	0	254.17	0
Janis_26w	F	dog	2014	Moving sheet	60.585	156.475	0
Lennon_6w	M	dog	2014	Rolled up mattress	25.589	3.598	19.737
Lennon_10w	M	dog	2014	Wheelbarrow	16.628	29.413	19.729
Lennon_14w	M	dog	2014	Mirror	15.913	54.314	0
Lennon_18w	M	dog	2014	Stuffed wolverine	21.243	4.843	42.025
Lennon_22w	M	dog	2014	Mechanical dog	11.504	159.205	0
Lennon_26w	M	dog	2014	Moving sheet	11.572	22.247	3.574
Marley_6w	M	dog	2014	Rolled up mattress	28.422	15.309	7.957
Marley_10w	M	dog	2014	Wheelbarrow	44.044	7.647	0
Marley_14w	M	dog	2014	Mirror	39.131	3.065	1.011
Marley_18w	M	dog	2014	Stuffed wolverine	0	287.018	0
Marley_22w	M	dog	2014	Mechanical dog	0	327.468	0
Marley_26w	M	dog	2014	Moving sheet	7.697	333.993	1.54

Table S2, continued

Individual	Sex	Species	Litter	Novel object	Invest_NO	Look_NO	Manip_NO
Björk_6w	F	wolf	2014	Rolled up mattress	24.939	7.938	0
Björk_10w	F	wolf	2014	Wheelbarrow	30.704	0	17.413
Björk_14w	F	wolf	2014	Mirror	67.582	3.071	14.622
Björk_18w	F	wolf	2014	Stuffed wolverine	14.082	1.027	5.11
Björk_22w	F	wolf	2014	Mechanical dog	13.722	5.399	3.343
Björk_26w	F	wolf	2014	Moving sheet	10.719	25.478	2.054
Iggy_6w	M	wolf	2014	Rolled up mattress	28.953	2.335	12.277
Iggy_10w	M	wolf	2014	Wheelbarrow	23.042	1.267	10.744
Iggy_14w	M	wolf	2014	Mirror	38.171	4.642	12.507
Iggy_18w	M	wolf	2014	Stuffed wolverine	22.75	2.778	71.645
Iggy_22w	M	wolf	2014	Mechanical dog	19.977	71.929	7.172
Iggy_26w	M	wolf	2014	Moving sheet	6.9	42.967	67.567
Joni_6w	F	wolf	2014	Rolled up mattress	42.269	0	13.576
Joni_10w	F	wolf	2014	Wheelbarrow	113.923	31	66.565
Joni_14w	F	wolf	2014	Mirror	43.519	5.369	13.05
Joni_18w	F	wolf	2014	Stuffed wolverine	10.241	8.647	163.715
Joni_22w	F	wolf	2014	Mechanical dog	37.632	6.645	265.54
Joni_26w	F	wolf	2014	Moving sheet	1.295	208.822	25.069
Lita_6w	F	wolf	2014	Rolled up mattress	36.645	5.01	0
Lita_10w	F	wolf	2014	Wheelbarrow	37.869	3.097	10.243
Lita_14w	F	wolf	2014	Mirror	21.78	8.702	5.139
Lita_18w	F	wolf	2014	Stuffed wolverine	11.534	4.133	45
Lita_22w	F	wolf	2014	Mechanical dog	20.482	3.698	14.848
Lita_26w	F	wolf	2014	Moving sheet	19.64	15.633	31.27
Ozzy_6w	M	wolf	2014	Rolled up mattress	42.991	4.608	4.118
Ozzy_10w	M	wolf	2014	Wheelbarrow	22.548	3.598	0
Ozzy_14w	M	wolf	2014	Mirror	28.69	10.57	0
Ozzy_18w	M	wolf	2014	Stuffed wolverine	7.977	2.298	42.982
Ozzy_22w	M	wolf	2014	Mechanical dog	2.039	17.907	191.145
Ozzy_26w	M	wolf	2014	Moving sheet	12.282	72.352	2.301
Billie_6w	F	dog	2015	Rolled up mattress	35.036	1.269	236.509
Billie_10w	F	dog	2015	Wheelbarrow	64.895	10.714	0
Billie_14w	F	dog	2015	Mirror	228.046	6.404	104.38
Billie_18w	F	dog	2015	Stuffed wolverine	12.892	8.7	155.144
Billie_22w	F	dog	2015	Mechanical dog		156.675	0
Billie_26w	F	dog	2015	Moving sheet	1.004	287.973	7.706
Ella_6w	F	dog	2015	Rolled up mattress	43.391	12.308	0
Ella_10w	F	dog	2015	Wheelbarrow	83.723	12.464	0
Ella_14w	F	dog	2015	Mirror	277.401	2.037	57.007
Ella_18w	F	dog	2015	Stuffed wolverine	13.086	16.419	86.028
Ella_22w	F	dog	2015	Mechanical dog	13.302	7.408	1.772
Ella_26w	F	dog	2015	Moving sheet	2.031	21.754	15.08

Table S2, continued

Individual	Sex	Species	Litter	Novel object	Invest_NO	Look_NO	Manip_NO
Muddy_6w	M	dog	2015	Rolled up mattress	64.642	0.507	189.218
Muddy_10w	M	dog	2015	Wheelbarrow	71.701	30.783	3.071
Muddy_14w	M	dog	2015	Mirror	184.929	9.733	50.667
Muddy_18w	M	dog	2015	Stuffed wolverine	15.357	3.596	18.95
Muddy_22w	M	dog	2015	Mechanical dog	8.17	33.063	0
Muddy_26w	M	dog	2015	Moving sheet	11.05	289.288	2.038
Red_6w	M	dog	2015	Rolled up mattress	56.028	6.414	0
Red_10w	M	dog	2015	Wheelbarrow	38.345	7.431	0
Red_14w	M	dog	2015	Mirror	104.427	1.267	15.108
Red_18w	M	dog	2015	Stuffed wolverine	8.182	0	599.183
Red_22w	M	dog	2015	Mechanical dog	12.549	2.175	486.048
Red_26w	M	dog	2015	Moving sheet	1.794	12.984	9.943
Simone_6w	F	dog	2015	Rolled up mattress	114.982	0.501	100.587
Simone_10w	F	dog	2015	Wheelbarrow	86.153	1.533	27.381
Simone_14w	F	dog	2015	Mirror	135.977	7.116	71.895
Simone_18w	F	dog	2015	Stuffed wolverine	6.137	0	37.77
Simone_22w	F	dog	2015	Mechanical dog	10.222	2.033	0
Simone_26w	F	dog	2015	Moving sheet	3.568	13.074	3.331
Skip_6w	M	dog	2015	Rolled up mattress	95.184	1.03	44
Skip_10w	M	dog	2015	Wheelbarrow	98.82	37.07	0
Skip_14w	M	dog	2015	Mirror	266.683	5.371	161.066
Skip_18w	M	dog	2015	Stuffed wolverine	35.395	12.328	0
Skip_22w	M	dog	2015	Mechanical dog	13.784	5.591	0
Skip_26w	M	dog	2015	Moving sheet	41.025	57.392	4.12
Flea_6w	M	wolf	2015	Rolled up mattress	86.793	14.762	31.947
Flea_10w	M	wolf	2015	Wheelbarrow	14.101	115.271	0
Flea_14w	M	wolf	2015	Mirror	107.52	38.597	9.999
Flea_18w	M	wolf	2015	Stuffed wolverine	22.502	69.188	4.121
Flea_22w	M	wolf	2015	Mechanical dog	24.786	60.259	0
Flea_26w	M	wolf	2015	Moving sheet	0.766	20.149	14.346
Hendrix_6w	M	wolf	2015	Rolled up mattress	10.238	0	52.93
Hendrix_10w	M	wolf	2015	Wheelbarrow	51.255	4.338	0
Hendrix_14w	M	wolf	2015	Mirror	75.441	13.077	36.64
Hendrix_18w	M	wolf	2015	Stuffed wolverine	15.321	1.798	12.602
Hendrix_22w	M	wolf	2015	Mechanical dog	11.24	4.5	0
Hendrix_26w	M	wolf	2015	Moving sheet	14.572	13.285	36.652
Elvis_6w	M	wolf	2016	Rolled up mattress	70.845	22.34	21.968
Elvis_10w	M	wolf	2016	Wheelbarrow	6.903	74.034	0
Elvis_14w	M	wolf	2016	Mirror	110.824	2.664	238.136
Elvis_18w	M	wolf	2016	Stuffed wolverine	48.436	8.52	7.411
Elvis_22w	M	wolf	2016	Mechanical dog	33.523	3.025	34.37
Elvis_26w	M	wolf	2016	Moving sheet	0	18.881	14.103

Table S2, continued

Individual	Sex	Species	Litter	Novel object	Invest_NO	Look_NO	Manip_NO
KD_6w	F	wolf	2016	Rolled up mattress	73.791	29.971	85.252
KD_10w	F	wolf	2016	Wheelbarrow	65.794	69.81	14.842
KD_14w	F	wolf	2016	Mirror	193.343	12.792	127.961
KD_18w	F	wolf	2016	Stuffed wolverine	20.433	10.788	42.345
KD_22w	F	wolf	2016	Mechanical dog	34.494	38.522	7.291
KD_26w	F	wolf	2016	Moving sheet	15.489	73.536	6.357
Lemmy_6w	M	wolf	2016	Rolled up mattress	85.933	6.909	101.883
Lemmy_10w	M	wolf	2016	Wheel barrel		95.109	
Moby_6w	M	wolf	2016	Rolled up mattress	46.788	31.431	107.534
Moby_10w	M	wolf	2016	Wheelbarrow	130.249	45.235	0
Moby_14w	M	wolf	2016	Mirror	116.22	3.739	165.807
Moby_18w	M	wolf	2016	Stuffed wolverine	0	240.913	0
Moby_22w	M	wolf	2016	Mechanical dog	17.672	24.973	0
Moby_26w	M	wolf	2016	Moving sheet	0	31.539	13.52
PJ_6w	F	wolf	2016	Rolled up mattress	30.463	0	0
PJ_10w	F	wolf	2016	Wheelbarrow	33.27	14.137	22.518
PJ_14w	F	wolf	2016	Mirror	208.021	0	212.876
PJ_18w	F	wolf	2016	Stuffed wolverine	7.582	0	382.116
PJ_22w	F	wolf	2016	Mechanical dog	6.915	4.728	69.374
PJ_26w	F	wolf	2016	Moving sheet	0	10.529	10.961
Sting_6w	M	wolf	2016	Rolled up mattress	99.796	8.515	55.313
Sting_10w	M	wolf	2016	Wheelbarrow	2.357	115.746	0
Sting_14w	M	wolf	2016	Mirror	125.396	0.416	262.587
Sting_18w	M	wolf	2016	Stuffed wolverine	22.601	10.231	28.041
Sting_22w	M	wolf	2016	Mechanical dog	7.922	7.904	4.453
Sting_26w	M	wolf	2016	Moving sheet	0.315	13.486	9.644

Table S3. Behavioural scores, non-novel object related. Scores (seconds) for behaviours not related to the novel object for each of the six novel object tests.

Individual	Sex	Species	Litter	Novel object	Active	Passive
Bowie_6w	M	dog	2014	Rolled up mattress	142.296	389.466
Bowie_10w	M	dog	2014	Wheelbarrow	301.909	211.311
Bowie_14w	M	dog	2014	Mirror	280.560	269.010
Bowie_18w	M	dog	2014	Stuffed wolverine	251.085	282.194
Bowie_22w	M	dog	2014	Mechanical dog	366.421	169.383
Bowie_26w	M	dog	2014	Moving sheet	250.25	260.526
Cash_6w	M	dog	2014	Rolled up mattress	227.865	322.020
Cash_10w	M	dog	2014	Wheelbarrow	203.605	350.518
Cash_14w	M	dog	2014	Mirror	234.937	246.965
Cash_18w	M	dog	2014	Stuffed wolverine	275.517	282.37
Cash_22w	M	dog	2014	Mechanical dog	435.587	140.379
Cash_26w	M	dog	2014	Moving sheet	308.122	244.775
Jagger_6w	M	dog	2014	Rolled up mattress	224.256	310.453
Jagger_10w	M	dog	2014	Wheelbarrow	114.892	434.056
Jagger_14w	M	dog	2014	Mirror	80.983	266.718
Jagger_18w	M	dog	2014	Stuffed wolverine	343.138	232.931
Jagger_22w	M	dog	2014	Mechanical dog	165.902	273.522
Jagger_26w	M	dog	2014	Moving sheet	22.282	510.208
Janis_6w	F	dog	2014	Rolled up mattress	157.952	407.461
Janis_10w	F	dog	2014	Wheelbarrow	187.159	373.693
Janis_14w	F	dog	2014	Mirror	175.78	345.231
Janis_18w	F	dog	2014	Stuffed wolverine	154.308	278.274
Janis_22w	F	dog	2014	Mechanical dog	87.341	206.164
Janis_26w	F	dog	2014	Moving sheet	182.345	165.554
Lennon_6w	M	dog	2014	Rolled up mattress	216.505	337.187
Lennon_10w	M	dog	2014	Wheelbarrow	193.06	343.918
Lennon_14w	M	dog	2014	Mirror	232.568	289.471
Lennon_18w	M	dog	2014	Stuffed wolverine	228.667	318.318
Lennon_22w	M	dog	2014	Mechanical dog	252.897	50.351
Lennon_26w	M	dog	2014	Moving sheet	264.822	289.439
Marley_6w	M	dog	2014	Rolled up mattress	251.358	299.438
Marley_10w	M	dog	2014	Wheelbarrow	215.824	353.716
Marley_14w	M	dog	2014	Mirror	98.789	464.741
Marley_18w	M	dog	2014	Stuffed wolverine	32.33	243.667
Marley_22w	M	dog	2014	Mechanical dog	145.401	125.077
Marley_26w	M	dog	2014	Moving sheet	23.066	224.249

Table S3, continued

Individual	Sex	Species	Litter	Novel object	Active	Passive
Björk_6w	F	wolf	2014	Rolled up mattress	309.287	236.074
Björk_10w	F	wolf	2014	Wheelbarrow	330.614	215.092
Björk_14w	F	wolf	2014	Mirror	333.41	198.047
Björk_18w	F	wolf	2014	Stuffed wolverine	411.962	177.738
Björk_22w	F	wolf	2014	Mechanical dog	391.398	181.024
Björk_26w	F	wolf	2014	Moving sheet	346.281	130.024
Iggy_6w	M	wolf	2014	Rolled up mattress	315.331	235.283
Iggy_10w	M	wolf	2014	Wheelbarrow	361.502	209.256
Iggy_14w	M	wolf	2014	Mirror	371.669	185.999
Iggy_18w	M	wolf	2014	Stuffed wolverine	319.042	202.75
Iggy_22w	M	wolf	2014	Mechanical dog	299.115	155.695
Iggy_26w	M	wolf	2014	Moving sheet	210.961	79.845
Joni_6w	F	wolf	2014	Rolled up mattress	446.995	63.484
Joni_10w	F	wolf	2014	Wheelbarrow	320.604	64.579
Joni_14w	F	wolf	2014	Mirror	456.491	118.725
Joni_18w	F	wolf	2014	Stuffed wolverine	280.531	97.422
Joni_22w	F	wolf	2014	Mechanical dog	251.098	41.656
Joni_26w	F	wolf	2014	Moving sheet	225.494	57.851
Lita_6w	F	wolf	2014	Rolled up mattress	317.281	254.176
Lita_10w	F	wolf	2014	Wheelbarrow	367.552	133.813
Lita_14w	F	wolf	2014	Mirror	328.241	247.411
Lita_18w	F	wolf	2014	Stuffed wolverine	472.924	81.347
Lita_22w	F	wolf	2014	Mechanical dog	414.14	134.121
Lita_26w	F	wolf	2014	Moving sheet	113.098	54.285
Ozzy_6w	M	wolf	2014	Rolled up mattress	309.207	217.584
Ozzy_10w	M	wolf	2014	Wheelbarrow	354.186	231.048
Ozzy_14w	M	wolf	2014	Mirror	510.317	68.619
Ozzy_18w	M	wolf	2014	Stuffed wolverine	473.281	66.293
Ozzy_22w	M	wolf	2014	Mechanical dog	29.697	0
Ozzy_26w	M	wolf	2014	Moving sheet	328.035	202.478
Billie_6w	F	dog	2015	Rolled up mattress	175.355	165.004
Billie_10w	F	dog	2015	Wheelbarrow	200.115	322.793
Billie_14w	F	dog	2015	Mirror	129.36	133.17
Billie_18w	F	dog	2015	Stuffed wolverine	147.751	283.102
Billie_22w	F	dog	2015	Mechanical dog	272.825	149.434
Billie_26w	F	dog	2015	Moving sheet	87.424	230.613
Ella_6w	F	dog	2015	Rolled up mattress	226.538	323.645
Ella_10w	F	dog	2015	Wheelbarrow	334.23	166.245
Ella_14w	F	dog	2015	Mirror	137.554	74.593
Ella_18w	F	dog	2015	Stuffed wolverine	268.578	190.812
Ella_22w	F	dog	2015	Mechanical dog	437.413	120.119
Ella_26w	F	dog	2015	Moving sheet	252.097	306.013

Table S3, continued

Individual	Sex	Species	Litter	Novel object	Active	Passive
Muddy_6w	M	dog	2015	Rolled up mattress	183.985	172.007
Muddy_10w	M	dog	2015	Wheelbarrow	302.825	204.557
Muddy_14w	M	dog	2015	Mirror	271.787	86.725
Muddy_18w	M	dog	2015	Stuffed wolverine	304.048	268.572
Muddy_22w	M	dog	2015	Mechanical dog	347.387	229.627
Muddy_26w	M	dog	2015	Moving sheet	137.575	118.116
Red_6w	M	dog	2015	Rolled up mattress	132.019	408.392
Red_10w	M	dog	2015	Wheelbarrow	181.521	381.893
Red_14w	M	dog	2015	Mirror	226.518	248.825
Red_18w	M	dog	2015	Stuffed wolverine	0	0
Red_22w	M	dog	2015	Mechanical dog	27.054	88.615
Red_26w	M	dog	2015	Moving sheet	365.456	212.435
Simone_6w	F	dog	2015	Rolled up mattress	124.314	276.682
Simone_10w	F	dog	2015	Wheelbarrow	358.881	127.5
Simone_14w	F	dog	2015	Mirror	186.052	187.158
Simone_18w	F	dog	2015	Stuffed wolverine	387.862	189.129
Simone_22w	F	dog	2015	Mechanical dog	499.094	102.357
Simone_26w	F	dog	2015	Moving sheet	360.406	226.171
Skip_6w	M	dog	2015	Rolled up mattress	126.445	332.59
Skip_10w	M	dog	2015	Wheelbarrow	194.366	240.694
Skip_14w	M	dog	2015	Mirror	110.092	65.243
Skip_18w	M	dog	2015	Stuffed wolverine	316.883	247.807
Skip_22w	M	dog	2015	Mechanical dog	457.673	137.407
Skip_26w	M	dog	2015	Moving sheet	274.248	227.882
Flea_6w	M	wolf	2015	Rolled up mattress	312.501	132.463
Flea_10w	M	wolf	2015	Wheelbarrow	251.697	124.757
Flea_14w	M	wolf	2015	Mirror	285.102	138.143
Flea_18w	M	wolf	2015	Stuffed wolverine	345.721	116.481
Flea_22w	M	wolf	2015	Mechanical dog	377.67	114.948
Flea_26w	M	wolf	2015	Moving sheet	32.511	15.909
Hendrix_6w	M	wolf	2015	Rolled up mattress	138.618	390.782
Hendrix_10w	M	wolf	2015	Wheelbarrow	218.726	305.184
Hendrix_14w	M	wolf	2015	Mirror	244.397	233.58
Hendrix_18w	M	wolf	2015	Stuffed wolverine	330.623	259.577
Hendrix_22w	M	wolf	2015	Mechanical dog	473.868	178.163
Hendrix_26w	M	wolf	2015	Moving sheet	283.79	134.824
Elvis_6w	M	wolf	2016	Rolled up mattress	244.733	250.799
Elvis_10w	M	wolf	2016	Wheelbarrow	342.56	170.195
Elvis_14w	M	wolf	2016	Mirror	188.81	75.914
Elvis_18w	M	wolf	2016	Stuffed wolverine	370.331	181.448
Elvis_22w	M	wolf	2016	Mechanical dog	384.406	141.025
Elvis_26w	M	wolf	2016	Moving sheet	48.049	34.143

Table S3, continued

Individual	Sex	Species	Litter	Novel object	Active	Passive
KD_6w	F	wolf	2016	Rolled up mattress	289.904	118.009
KD_10w	F	wolf	2016	Wheelbarrow	289.217	115.959
KD_14w	F	wolf	2016	Mirror	159.677	133.547
KD_18w	F	wolf	2016	Stuffed wolverine	292.995	217.81
KD_22w	F	wolf	2016	Mechanical dog	361.189	158.362
KD_26w	F	wolf	2016	Moving sheet	307.914	54.177
Lemmy_6w	M	wolf	2016	Rolled up mattress	304.722	100.247
Lemmy_10w	M	wolf	2016	Wheelbarrow	300.52	72.63
Moby_6w	M	wolf	2016	Rolled up mattress	236.52	194.079
Moby_10w	M	wolf	2016	Wheelbarrow	294.773	63.035
Moby_14w	M	wolf	2016	Mirror	199.795	127.798
Moby_18w	M	wolf	2016	Stuffed wolverine	246.043	153.462
Moby_22w	M	wolf	2016	Mechanical dog	367.147	187.665
Moby_26w	M	wolf	2016	Moving sheet	31.096	24.753
PJ_6w	F	wolf	2016	Rolled up mattress	367.021	180.968
PJ_10w	F	wolf	2016	Wheelbarrow	399.582	110.937
PJ_14w	F	wolf	2016	Mirror	113.377	67.835
PJ_18w	F	wolf	2016	Stuffed wolverine	130.596	80.988
PJ_22w	F	wolf	2016	Mechanical dog	269.428	74.316
PJ_26w	F	wolf	2016	Moving sheet	259.093	183.891
Sting_6w	M	wolf	2016	Rolled up mattress	280.838	149.282
Sting_10w	M	wolf	2016	Wheelbarrow	361.584	95.797
Sting_14w	M	wolf	2016	Mirror	145.668	76.903
Sting_18w	M	wolf	2016	Stuffed wolverine	406.636	135.894
Sting_22w	M	wolf	2016	Mechanical dog	473.474	99.104
Sting_26w	M	wolf	2016	Moving sheet	109.991	53.29

Table S4. Fear behaviours. Scores (frequency) of fear behaviours expressed in relation to the novel object for all tests at six, 10, 14, 18, 22 and 26 weeks (w) of age. Empty cells indicate 0 counts.

Individual	Species	Fleeing	Growl	Low body	Pilo-erection	Retreat	Startle	Tugged tail
Bowie_6w	dog							
Bowie_10w	dog							
Bowie_14w	dog							
Bowie_18w	dog			1	2			
Bowie_22w	dog							
Bowie_26w	dog	1		1		1	2	
Cash_6w	dog							
Cash_10w	dog							
Cash_14w	dog			1				
Cash_18w	dog							
Cash_22w	dog			1				
Cash_26w	dog							
Jagger_6w	dog			1				
Jagger_10w	dog							
Jagger_14w	dog							
Jagger_18w	dog							
Jagger_22w	dog			12		10	9	
Jagger_26w	dog					1	2	
Janis_6w	dog							
Janis_10w	dog			1				
Janis_14w	dog							
Janis_18w	dog			4	5	4		
Janis_22w	dog			17		13	1	
Janis_26w	dog					2	4	
Lennon_6w	dog							
Lennon_10w	dog							
Lennon_14w	dog							
Lennon_18w	dog			1	1			
Lennon_22w	dog					6	6	
Lennon_26w	dog						1	
Marley_6w	dog							
Marley_10w	dog							
Marley_14w	dog							
Marley_18w	dog	1	5		2	1	1	
Marley_22w	dog				2	1	5	
Marley_26w	dog						5	

Table S4 continued

Individual	Species	Fleeing	Growl	Low body	Pilo-erection	Retreat	Startle	Tugged tail
Björk_6w	wolf							
Björk_10w	wolf							
Björk_14w	wolf			2				
Björk_18w	wolf							
Björk_22w	wolf							
Björk_26w	wolf			2			1	
Iggy_6w	wolf							
Iggy_10w	wolf							
Iggy_14w	wolf							
Iggy_18w	wolf			1				
Iggy_22w	wolf			8		3	2	
Iggy_26w	wolf						2	
Joni_6w	wolf			1				
Joni_10w	wolf			6		4		
Joni_14w	wolf							
Joni_18w	wolf			4		1		
Joni_22w	wolf			2				
Joni_26w	wolf		2	12	2	3	15	
Lita_6w	wolf							
Lita_10w	wolf							
Lita_14w	wolf							
Lita_18w	wolf			2		1		
Lita_22w	wolf	1		1		1	1	
Lita_26w	wolf							
Ozzy_6w	wolf							
Ozzy_10w	wolf			1		1		
Ozzy_14w	wolf			1				
Ozzy_18w	wolf							
Ozzy_22w	wolf			2		1	4	
Ozzy_26w	wolf	1		2			4	
Billie_6w	dog							
Billie_10w	dog							
Billie_14w	dog							
Billie_18w	dog			2		1		
Billie_22w	dog	1		6		6	2	
Billie_26w	dog						5	
Ella_6w	dog							
Ella_10w	dog					1		
Ella_14w	dog							
Ella_18w	dog		2	3		8		1
Ella_22w	dog	1		6		2	4	
Ella_26w	dog						1	

Table S4 continued

Individual	Species	Fleeing	Growl	Low body	Pilo-erection	Retreat	Startle	Tugged tail
Muddy_6w	dog							
Muddy_10w	dog							
Muddy_14w	dog							
Muddy_18w	dog							
Muddy_22w	dog			2			2	
Muddy_26w	dog		1	1		2	12	
Red_6w	dog							
Red_10w	dog					3		
Red_14w	dog							
Red_18w	dog			1		1		
Red_22w	dog			3			1	
Red_26w	dog							
Simone_6w	dog							
Simone_10w	dog							
Simone_14w	dog							
Simone_18w	dog							
Simone_22w	dog							
Simone_26w	dog						1	
Skip_6w	dog							
Skip_10w	dog					2		
Skip_14w	dog							
Skip_18w	dog							
Skip_22w	dog			2			2	
Skip_26w	dog			2		1		
Flea_6w	wolf			1				1
Flea_10w	wolf			10		8	1	
Flea_14w	wolf			2		1		
Flea_18w	wolf		1	7		2		
Flea_22w	wolf			3			5	
Flea_26w	wolf		1	1			2	
Hendrix_6w	wolf							
Hendrix_10w	wolf			1				
Hendrix_14w	wolf			1				
Hendrix_18w	wolf							
Hendrix_22w	wolf			1				
Hendrix_26w	wolf			1				1
Elvis_6w	wolf							
Elvis_10w	wolf			4		4		1
Elvis_14w	wolf			1				
Elvis_18w	wolf			3		1		1
Elvis_22w	wolf							
Elvis_26w	wolf			2		3	4	1

Table S4 continued

Individual	Species	Fleeing	Growl	Low body	Pilo-erection	Retreat	Startle	Tugged tail
KD_6w	wolf					1		
KD_10w	wolf			5	6	6		1
KD_14w	wolf			4		2		
KD_18w	wolf			5		5		2
KD_22w	wolf			4			1	
KD_26w	wolf	2		4		3	4	
Lemmy_6w	wolf							
Lemmy_10w	wolf				2	16		
Moby_6w	wolf			1				
Moby_10w	wolf			5		8	1	
Moby_14w	wolf							
Moby_18w	wolf			1	3			
Moby_22w	wolf			3			2	
Moby_26w	wolf			2		2	2	
PJ_6w	wolf							
PJ_10w	wolf					6		3
PJ_14w	wolf			1				3
PJ_18w	wolf			2				1
PJ_22w	wolf							1
PJ_26w	wolf			1		2	1	
Sting_6w	wolf							
Sting_10w	wolf				2	6	1	
Sting_14w	wolf			2			2	
Sting_18w	wolf			4		2		
Sting_22w	wolf			3		1		
Sting_26w	wolf	1		2			4	

Table S5. Random effects estimates. Estimates of the standard deviations for the intercept terms in the models used for all behavioural variables. Residual standard deviations are further included for all other behaviours than latency measures.

Behaviour	Group	Term	Std.dev
Latency, approach	individual	intercept	0.70727
	relatedness	intercept	0.02002
Latency, contact	individual	intercept	0.00679
	relatedness	intercept	0.00342
Looking at NO	relatedness	intercept	5.00E-05
	individual	intercept	0.6147
	Residual	intercept	3.30652
Investigating NO	relatedness	intercept	1.00E-05
	individual	intercept	5.00E-05
	Residual	intercept	3.13226
Manipulating NO	relatedness	intercept	0.13058
	individual	intercept	0.00013
	Residual	intercept	3.139
Active behaviour	individual	intercept	37.8062
	relatedness	intercept	0
	Residual	intercept	287.73584
Passive behaviour	individual	intercept	19.49431
	relatedness	intercept	47.76699
	Residual	intercept	235.7152

Table S6. Model selection. Model selection table for each behaviour, listing the degrees of freedom (df), Akaike's Information Criterion (AIC), the difference in AIC with the best model (delta), and which model was selected.

response_variable	method	model	df	AIC	delta
Latency_approach	coxme	species * age + sex	13.3	1142.61	0
Latency_approach	coxme	null	7.59	1155.97	13.36
Latency_contact	coxme	species * age + sex	4.01	1118.04	0
Latency_contact	coxme	null	0.02	1121.54	3.5
Looking at NO	gamlss, lognormal	species * age + sex + duration	16.98	11465.9	0
Looking at NO	gamlss, lognormal	duration	13.87	11910.53	444.63
Investigating NO	gamlss, lognormal	species * age + sex + duration	2	13316.79	0
Investigating NO	gamlss, lognormal	duration	2	13769.98	453.2
Manipulating NO	gamlss, lognormal	species * age + sex + duration	2.63	12320.42	0
Manipulating NO	gamlss, lognormal	duration	4.36	12345.89	25.47
Active behaviour	lmer	species * age + sex + duration	9	1732.79	0
Active behaviour	lmer	duration	5	1758.43	25.64
Passive behaviour	lmer	species * age + sex + duration	9	1673.39	0
Passive behaviour	lmer	duration	5	1707.85	34.47

Table S7. Anova table. Anova results for the best fitted model of repeated measures, with dogs as the reference, for each behaviour. Df, $\chi2$, F and p-value are given. Significant p-values are marked in bold italic.

response_variable	term	df	Chisq	F	p
latency_approach	species	1	2.671		0.102
	age_centered	1	145.946		***<0.0001***
	sex	1	2.021		0.155
	species:age_centered	1	66.286		***<0.0001***
latency_contact	species	1	1.585		0.208
	age_centered	1	7.803		***0.005***
	sex	1	0.079		0.778
	species:age_centered	1	2.763		0.096
Looking at NO	(Intercept)	1, 118		0.004	0.948
	species	1, 2		0.015	0.914
	age_centered	1, 118		33.991	***<0.0001***
	sex	1, 20		1.232	0.28
	duration	1, 118		0.663	0.417
	species:age_centered	1, 118		4.219	***0.042***
Investigating NO	(Intercept)	1, 118		0	1
	species	1, 2		0.424	0.582
	age_centered	1, 118		56.78	***<0.0001***
	sex	1, 20		0.524	0.478
	duration	1, 118		4.079	***0.046***
	species:age_centered	1, 118		3.723	0.056
Manipulating NO	(Intercept)	1, 118		0.014	0.905
	species	1, 2		2.647	0.245
	age_centered	1, 118		1.511	0.221
	sex	1, 20		2.467	0.132
	duration	1, 118		0.831	0.364
	species:age_centered	1, 118		1.978	0.162
Active behaviour	species	1, 23.96		21.405	***<0.0001***
	age_centered	1, 121.98		4.472	***0.036***
	sex	1, 22.44		0.146	0.706
	duration	1, 124.13		11.643	***0.001***
	species:age_centered	1, 121.93		0.018	0.894
Passive behaviour	species	1, 2		2.553	0.251
	age_centered	1, 120.98		16.252	***<0.0001***
	sex	1, 19.41		0.092	0.765
	duration	1, 124.27		1.912	0.169
	species:age_centered	1, 120.92		1.586	0.21

Table S8. Post hoc testing. Results from Cox regression, estimating the marginal effects of species at each age point in. Estimate, standard error, test statistic (z or t) and p-values and adjusted p-values are given. P-values were adjusted for multiple testing using Holm's method. Significant p-values are marked in bold italic

Behaviour	Contrast	Age	Estimate	SE	z	t	p	adjusted p
Latency, approach	dog-wolf	6 weeks	-0.142	0.351	-0.403		0.687	1
	dog-wolf	10 weeks	0.105	0.277	0.38		0.704	1
	dog-wolf	14 weeks	0.352	0.234	1.51		0.132	0.395
	dog-wolf	18 weeks	0.599	0.239	2.51		***0.012***	***0.048***
	dog-wolf	22 weeks	0.846	0.29	2.92		***0.003***	***0.018***
	dog-wolf	26 weeks	1.09	0.368	2.97		***0.003***	***0.018***
Looking at NO	dog-wolf	6 weeks	-0.708	0.436		-1.62	0.246	1
	dog-wolf	10 weeks	-0.446	0.365		-1.22	0.346	1
	dog-wolf	14 weeks	-0.185	0.33		-0.562	0.631	1
	dog-wolf	18 weeks	0.0761	0.341		0.223	0.844	1
	dog-wolf	22 weeks	0.337	0.396		0.852	0.484	1
	dog-wolf	26 weeks	0.599	0.479		1.25	0.338	1

Table S9. Age trends. Estimated marginal means of the age trends for dogs of wolves. Slope estimate, standard error, df and lower and upper 95% CI's are given for each species.

Species	Slope estimate	SE	df	95CI low	95CI up
Dog	0.1065	0.0266	Inf.	0.05425	0.1587
Wolf	0.0192	0.0134	Inf.	-0.00695	0.0454

Table S10. Model selection, excluding week 14. Model selection table for each behaviour for models excluding week 14. Listed are the degrees of freedom (df), Akaike's Information Criterion (AIC), the difference in AIC with the best model (delta), and which model was selected.

response_variable	method	model	df	AIC	delta
latency_approach	coxme	species * age + sex	14.34	904.72	0
latency_approach	coxme	null	7.91	921.9	17.17
latency_contact	coxme	species * age + sex	4	886.3	0
latency_contact	coxme	species + age + sex	3	887.08	0.78
latency_contact	coxme	null	0.03	887.78	1.48
look_at_object	gamlss, lognormal	species * age + sex + duration	17.42	9800.55	0
look_at_object	gamlss, lognormal	duration	13.82	10229.44	428.89
investigate_object	gamlss, lognormal	species * age + sex + duration	2.17	10108.97	0
investigate_object	gamlss, lognormal	duration	2	10569.15	460.18
manipulate_object	gamlss, lognormal	species * age + sex + duration	2	9711.61	0
manipulate_object	gamlss, lognormal	duration	3.35	9752.88	41.26
active	lmer	species * age + sex + duration	9	1437.25	0
active	lmer	duration	5	1462.68	25.43
passive	lmer	species * age + sex + duration	9	1392.44	0
passive	lmer	duration	5	1429.59	37.15

Table S11. Model summary, excluding week 14. Results for the best fitted model of repeated measures, with dogs as the reference, for each behaviour excluding data from week 14 (the mirror novel object). Estimate, standard error, degrees of freedom, t-value and p-value are given. Significant p-values are marked in bold italic. Base level (intercept) for the models is dog at week 16 (the centered age 0).

response_variable	term	estimate	se	z	t	p
Latency approach	specieswolf	-0.537	0.264	-2.03	NA	***0.042***
	age_centered	0.094	0.022	4.21	NA	***<0.0001***
	sexMale	0.413	0.278	1.48	NA	*0.14*
	specieswolf:age_centered	-0.066	0.028	-2.32	NA	***0.02***
Looking at NO	(Intercept)	-1.182	1.347	NA	-0.877	0.383
	specieswolf	0.087	0.37	NA	0.237	0.835
	age_centered	0.116	0.021	NA	5.529	***<0.0001***
	sexMale	0.398	0.378	NA	1.053	0.305
	duration	0.09	0.127	NA	0.713	0.478
	specieswolf:age_centered	-0.058	0.032	NA	-1.832	0.07
Investigating_NO	(Intercept)	-1.852	1.06	NA	-1.748	0.084
	specieswolf	0.159	0.19	NA	0.835	0.492
	age_centered	-0.109	0.017	NA	-6.378	***<0.0001***
	sexMale	-0.069	0.19	NA	-0.363	0.721
	duration	0.182	0.102	NA	1.783	0.078
	specieswolf:age_centered	0.043	0.026	NA	1.669	0.098
Manipulating_NO	(Intercept)	2.027	1.916	NA	1.058	0.293
	specieswolf	0.397	0.342	NA	1.159	0.366
	age_centered	-0.057	0.031	NA	-1.852	0.067
	sexMale	-0.493	0.342	NA	-1.443	0.165
	duration	-0.19	0.184	NA	-1.031	0.305
	specieswolf:age_centered	0.06	0.047	NA	1.28	0.204
Active behaviour	(Intercept)	-135.066	95.18	NA	-1.419	0.159
	specieswolf	106.557	23.142	NA	4.604	***<0.0001***
	age_centered	2.386	1.497	NA	1.594	0.114
	sexMale	-14.692	23.548	NA	-0.624	0.539
	duration	36.514	9.023	NA	4.047	***<0.0001***
	specieswolf:age_centered	0.08	2.273	NA	0.035	0.972
Passive behaviour	(Intercept)	115.905	82.747	NA	1.401	0.166
	specieswolf	-86.268	40.335	NA	-2.139	0.173
	age_centered	-5.422	1.254	NA	-4.325	***<0.0001***
	sexMale	12.964	17.842	NA	0.727	0.476
	duration	12.325	7.532	NA	1.636	0.105
	specieswolf:age_centered	2.753	1.899	NA	1.45	0.15

Table S12. Anova table, excuding week 14. Anova results for the best fitted model of repeated measures, with dogs as the reference, for each behaviour excuding the mirror test at week 14. Df, $\chi 2$, F and p-value are given. Significant p-values are marked in bold italic.

response_variable	term	df	Chisq	F	p
latency_approach	species	1	3.946		***0.047***
	age_centered	1	14.387		***<0.0001***
	sex	1	2.205		0.138
	species:age_centered	1	5.386		***0.02***
latency_contact	species	1	1.15		0.283
	age_centered	1	6.16		***0.013***
	sex	1	0.085		0.771
	species:age_centered	1	2.786		0.095
Looking at NO	(Intercept)	1, 94		0.004	0.949
	species	1, 2		0.04	0.86
	age_centered	1, 94		30.995	***<0.0001***
	sex	1, 20		1.205	0.285
	duration	1, 94		1.471	0.228
	species:age_centered	1, 94		3.356	***0.07***
Investigating NO	(Intercept)	1, 94		0	1
	species	1, 2		0.989	0.425
	age_centered	1, 94		57.243	***<0.0001***
	sex	1, 20		0.245	0.626
	duration	1, 94		2.008	0.16
	species:age_centered	1, 94		2.787	0.098
Manipulating NO	(Intercept)	1, 94		0	1
	species	1, 2		2.648	0.245
	age_centered	1, 94		0.96	0.33
	sex	1, 20		2.14	0.159
	duration	1, 94		1.924	0.169
	species:age_centered	1, 94		1.637	0.204
Active behaviour	species	1, 24.71		21.201	***<0.0001***
	age_centered	1, 97.83		4.513	***0.036***
	sex	1, 22.8		0.389	0.539
	duration	1, 99.3		16.375	***<0.0001***
	species:age_centered	1, 97.8		0.001	0.972
Passive behaviour	species	1, 1.9		4.574	0.173
	age_centered	1, 96.43		17.968	***<0.0001***
	sex	1, 19.87		0.528	0.476
	duration	1, 98.72		2.678	0.105
	species:age_centered	1, 96.39		2.102	0.15

Table S13. Model selection, NO manipulation week 18, 22 and 26. Model selection table for each behaviour for models excluding week 14. Listed are the degrees of freedom (df), Akaike's Information Criterion (AIC), the difference in AIC with the best model (delta), and which model was selected.

response_variable	method	model	df	AIC	delta
manipulate_object	gamlss	species * age + sex + duration	16.69	5056.77	0
manipulate_object	gamlss	duration	12.48	5418.17	361.4

Table S14. Model output, NO manipulation week 18, 22 and 26. Results for the best fitted model of repeated measures, with dogs as the reference, for manipulation behaviour in wolves and dogs at 18, 22 and 26 weeks. Estimate, standard error, degrees of freedom, t-value and p-value are given. Significant p-values are marked in bold italic. Base level for the models is dog at week 16 (the centered age 0).

response_variable	term	estimate	se	t	p
**manipulate object (w 18, 22, 26)**	(Intercept)	5.603	1.695	3.305	***0.002***
**manipulate object (w 18, 22, 26)**	specieswolf	0.053	0.752	0.071	0.95
**manipulate object (w 18, 22, 26)**	age_centered	-0.285	0.065	-4.407	***<0.0001***
**manipulate object (w 18, 22, 26)**	sexMale	-0.874	0.517	-1.689	0.108
**manipulate object (w 18, 22, 26)**	duration	-0.366	0.151	-2.421	***0.02***
**manipulate object (w 18, 22, 26)**	specieswolf:age_centered	0.056	0.106	0.532	0.597

Table S15. Anova table, NO manipulation week 18, 22 and 26. Anova results for the best fitted model of repeated measures, with dogs as the reference, for for manipulating the novel object at 18, 22 and 26 weeks (w). Df, F and p-value are given. Significant p-values are marked in bold italic.

response_variable	term	df	F	p
manipulate object (w 18, 22, 26)	(Intercept)	1, 118	0.014	0.905
manipulate object (w 18, 22, 26)	species	1, 2	2.647	0.245
manipulate object (w 18, 22, 26)	age_centered	1, 118	1.511	0.221
manipulate object (w 18, 22, 26)	sex	1, 20	2.467	0.132
manipulate object (w 18, 22, 26)	duration	1, 118	0.831	0.364
manipulate object (w 18, 22, 26)	species:age_centered	1, 118	1.978	0.162

Figure S1. NO manipulation week 18, 22 and 26. Boxplots shows behavioural scores during novel object test, comparing manipulation of the novel object in dogs and wolves at 18, 22 and 26 weeks. Overlaid are the fits and confidence intervals from the best model, selected by AIC. Boxes indicate the quartiles, and the whiskers reach maximally 1.5 times the interquartile range. Values beyond that are shown as points. An log(y) scale) was used.

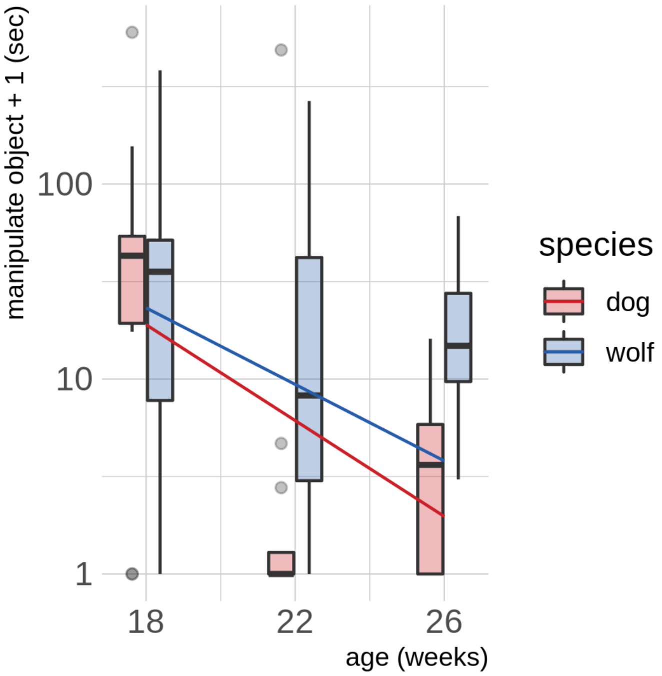

Supplement: Supplementary file 2 [file Data_Sheet_1.PDF]
